# The practice and promise of temporal genomics for measuring evolutionary responses to global change

**DOI:** 10.1111/1755-0998.13789

**Published:** 2023-04-02

**Authors:** René D. Clark, Katrina A. Catalano, Kyra S. Fitz, Eric Garcia, Kyle E. Jaynes, Brendan N. Reid, Allyson Sawkins, Anthony A. Snead, John C. Whalen, Malin L. Pinsky

**Affiliations:** ^1^ Department of Ecology, Evolution and Natural Resources Rutgers University New Brunswick New Jersey USA; ^2^ Department of Biological Sciences Old Dominion University Norfolk Virginia USA; ^3^ Department of Integrative Biology W.K. Kellogg Biological Station Michigan State University Hickory Corners Michigan USA; ^4^ Ecology, Evolution, and Behavior Program Michigan State University East Lansing Michigan USA; ^5^ Department of Ecology and Evolutionary Biology University of California Santa Cruz Santa Cruz California USA; ^6^ Department of Biological Sciences University of Alabama Tuscaloosa Alabama USA

**Keywords:** contemporary evolution, genetic monitoring, historical DNA

## Abstract

Understanding the evolutionary consequences of anthropogenic change is imperative for estimating long‐term species resilience. While contemporary genomic data can provide us with important insights into recent demographic histories, investigating past change using present genomic data alone has limitations. In comparison, temporal genomics studies, defined herein as those that incorporate time series genomic data, utilize museum collections and repeated field sampling to directly examine evolutionary change. As temporal genomics is applied to more systems, species and questions, best practices can be helpful guides to make the most efficient use of limited resources. Here, we conduct a systematic literature review to synthesize the effects of temporal genomics methodology on our ability to detect evolutionary changes. We focus on studies investigating recent change within the past 200 years, highlighting evolutionary processes that have occurred during the past two centuries of accelerated anthropogenic pressure. We first identify the most frequently studied taxa, systems, questions and drivers, before highlighting overlooked areas where further temporal genomic studies may be particularly enlightening. Then, we provide guidelines for future study and sample designs while identifying key considerations that may influence statistical and analytical power. Our aim is to provide recommendations to a broad array of researchers interested in using temporal genomics in their work.

## 1 INTRODUCTION

Given the acceleration of global change, characterizing the evolutionary impact of anthropogenic pressures on populations, species and communities is critical for estimating vulnerability and identifying potential conservation strategies imperative for long‐term persistence (Kinnison & Hairston, 2007). The modern integration of molecular tools in ecology, evolution and conservation has brought an influx of studies assessing how organisms respond to large‐scale human‐mediated change. For example, recent objectives include identifying genomic signatures of adaptation, shifts in population connectivity, reductions in population size and changes in levels of genetic diversity (e.g., Athrey et al., 2012; Campbell‐Staton et al., 2020; Leigh et al., 2019). Most of these studies use present‐day genomic patterns and data to infer recent demographic histories and assess their implications for future resilience (Beichman et al., 2018).

Although genomic data from contemporary populations provide us with important insights, investigating past events with present‐day data alone is often quite complicated (Jensen & Leigh, 2022). Evolutionary studies using samples from a single time point can indirectly infer ancestral states using coalescent‐based models (Dehasque et al., 2020). However, assumptions for these models are often unrealistic and can cause difficulties when parsing the impact of different evolutionary drivers (Buffalo & Coop, 2020; Dehasque et al., 2020). Furthermore, the lack of a historical baseline restricts our ability to estimate recent changes in population size, connectivity, adaptive potential and bouts of selection (Nielsen & Hansen, 2008; Snead & Clark, 2022). In comparison, temporal genomics studies, defined herein as studies that incorporate time series genomic data, harness the power of museum collections or repeated field sampling to uncover the evolutionary consequences of past events. Sampling a population multiple times can provide benefits including, but not limited to, (i) direct measurements of allele frequency change over time, (ii) information to disentangle the role of genetic drift and selection in allele frequency changes (Buffalo & Coop, 2019, 2020), (iii) substantially better inference of selection strength, particularly in polygenic cases (Buffalo & Coop, 2020; Foll et al., 2015), (iv) opportunities to correlate allele frequencies with long‐term environmental variation (Czorlich et al., 2022), (v) more accurate identification of immigrants and their influence on long‐term effective population size (*N*_e_) estimates (García‐Navas et al., 2015; Gilbert & Whitlock, 2015) and (vi) improved ability to separate mapping, bioinformatic or sampling errors from signal.

While we have had the ability to conduct temporal genomics analyses for at least the past few decades (Pääbo, 1989), high sequencing costs and issues with degraded DNA have largely made temporal studies impractical to conduct with regularity. Advancements in DNA extraction, amplification and library preparation protocols (reviewed in Hofreiter et al., 2015), coupled with the recent increase in the availability of next‐generation sequencing, have made it possible to answer previously intractable questions, particularly on larger scales and in nonmodel systems (Bi et al., 2013; Card et al., 2021; Rowe et al., 2011). With this increased feasibility, researchers can now combine larger sample sizes and higher‐quality DNA with dense sampling across space and time, enabling us to approach important evolutionary questions from an entirely new perspective. For example, Czorlich et al. (2022) used archived scales to link the harvest of capelin (*Mallotus villosus*), a small marine fish, to its indirect effects driving evolutionary change in Atlantic salmon (*Salmo salar*), a common predator. Similarly, Popa‐Báez et al. (2020) combined contemporary data with those from past studies to characterize the genomic consequences of range expansion in populations of Queensland fruit flies (*Bactrocera tryoni*) and identify progressively reduced genetic diversity along one expansion route. Other recent studies have harnessed temporal methods to uncover land use change leading to historical declines in *N*_e_ (Lonsinger et al., 2018), habitat fragmentation causing losses of genetic diversity (Gauthier et al., 2020) and stochastic environmental variation contributing to temporal shifts in the genomic composition of populations (Therkildsen et al., 2013).

With this growth, the field of temporal genomics has also experienced a proliferation of sampling and statistical approaches with differing power and validity for inference (Lopez et al., 2020). As temporal genomic approaches are applied to additional systems, species and questions, best practices are essential to make powerful inferences and further our understanding of the evolutionary consequences of anthropogenic activities. To assess current practices, we conducted a systematic literature review to develop recommendations for detecting recent evolutionary change from temporal sampling. We focused on studies investigating change within the last 200 years, narrowing in on evolutionary processes during a period of accelerated anthropogenic pressure. We purposely excluded studies of plants and fungi, as these taxa differ from animals in evolutionarily important ways (e.g., polyploidy prevalence, dispersal modes and reproductive mechanisms), such that a comprehensive assessment encompassing all three kingdoms was beyond the scope of this review (but see Bieker & Martin, 2018). Similarly, we do not focus on ancient DNA research over deeper timescales (e.g., thousands of years), which is a related but distinct field. While previous review papers have mostly addressed museum best practices (Card et al., 2021) or focused on specific taxa (Billerman & Walsh, 2019), our review addresses study design issues applicable across laboratory techniques and systems. Specifically, we address how experimental design considerations impact the ability to detect temporal change.

Our review is divided into two main portions: a “State of the Field” section and a “Best Practices” section. In the “State of the Field” section, we present the empirical results of our literature review, identifying common study designs as well as which taxa, systems, questions and drivers are most frequently investigated. Then, in the “Best Practices” section, we discuss common pitfalls within temporal genomics, provide guidelines for future study designs and identify key considerations that may influence analytical power. We hope these suggestions will help guide the design of future studies with the goal of increasing the efficacy of temporal genomics to assess the current critical state of natural ecosystems.

## 2 STATE OF THE FIELD

### 2.1 Taxonomic and geographical trends in temporal genomics studies

We first set out to identify shared aspects of recent temporal genomics studies and key gaps in the field. To enable such a broad summary, we conducted a literature search using the keywords “temporal genomics,” “temporal genetics,” “historical DNA (hDNA),” “ancient DNA” and “museum DNA,” including their common variants (“aDNA,” “hDNA,” etc.). We retained studies from 2000 to 2020 that investigated genetic or genomic change in wild animal populations using temporal samples from no earlier than 1800. While the forward‐looking aspects of this review centre on genomic studies, our literature review includes many studies that would traditionally be considered “genetic” (e.g., limited to one or a few loci), to completely capture study trends as the field continues to transition into the genomics era. For a full overview of the search and inclusion criteria, see Methods S1.

**FIGURE 1:**
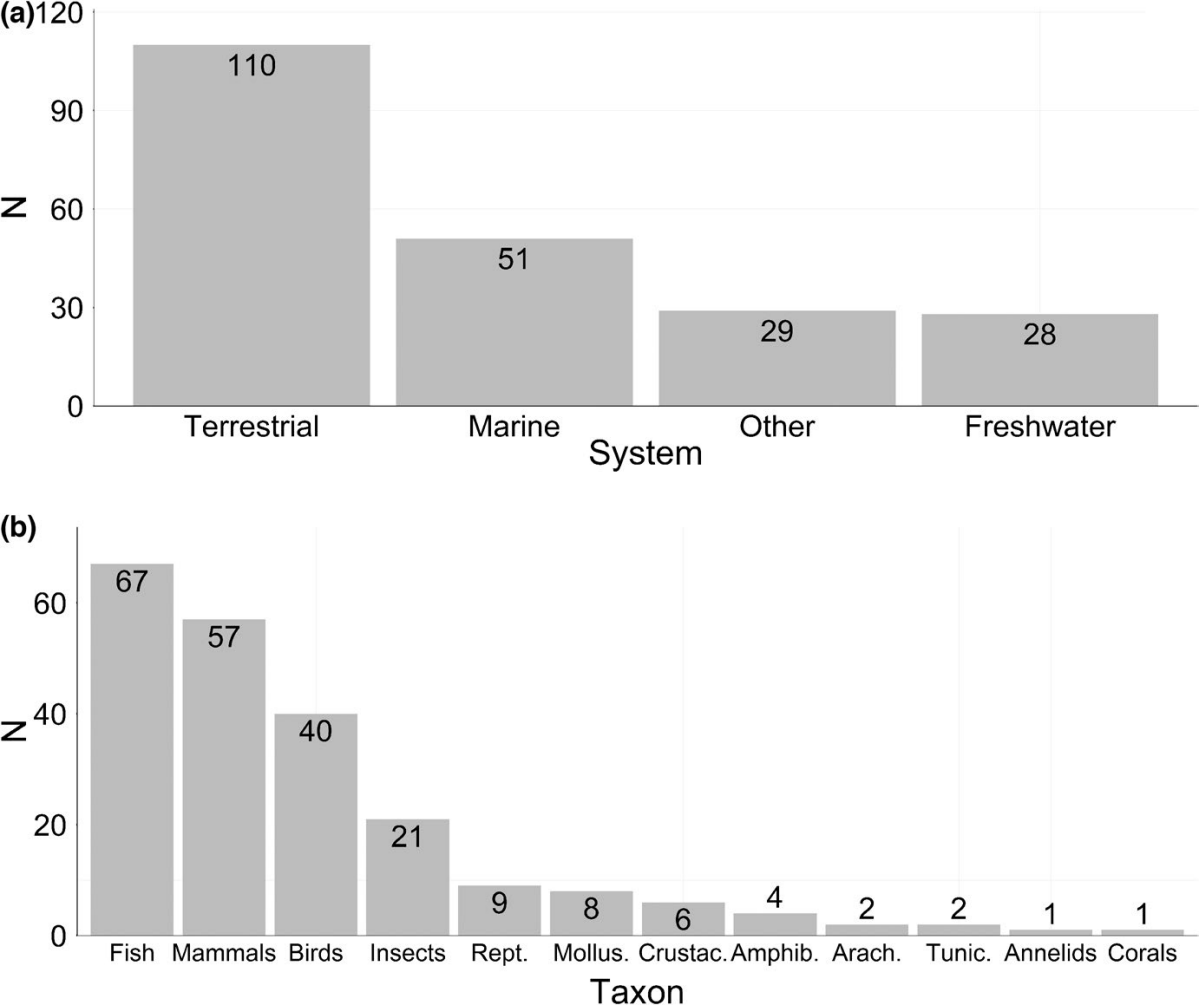
Distribution of temporal genomics studies across systems (a) and taxa (b). Numbers above/within bars represent counts per system/taxon. The 16 observed taxonomic classes were condensed into higher‐order taxa where appropriate. Amphib., amphibians; Arach., arachnids; Crustac., crustaceans; Mollus., molluscs; Rept., reptiles; Tunic., tunicates.

Of the 218 studies retained in our literature search, 51% investigated genetic change in terrestrial species, 23% in marine, 13% in freshwater and 13% in species that split their life cycle between at least two of the three realms (hereafter referred to as “other”; Figure 1a). This last category was primarily composed of anadromous and catadromous fishes, amphibians, waterfowl, marine mammals and invertebrates. While 16 taxonomic classes were represented in our data set, most studies focused on change in “charismatic” vertebrate organisms (e.g., mammals [26%] and birds [18%]), or in taxa with well‐documented histories of population monitoring, such as many fish stocks (31%; Figure 1b). Insects were the focus of 10% of the studies, while 4% looked at reptiles (Figure 1b). The remaining 11% comprised a variety of taxa, including amphibians, crustaceans, molluscs and coral (Figure 1b). The differences in the number of studies per system and taxonomic group may represent a disparity both in available historical samples (Meineke & Daru, 2021) as well as in the relative interest in, or notoriety of, major changes in each taxon (Wagner et al., 2021). Notably, there were far fewer studies investigating genetic change in short‐lived organisms (those with generation times of 1 year or less). As these organisms are often some of the first to respond to anthropogenic change (Foden et al., 2019), future temporal genomics studies on short‐lived species may provide particularly productive opportunities to identify genomic mechanisms underlying rapid adaptation to novel selective pressures.

**FIGURE 2:**
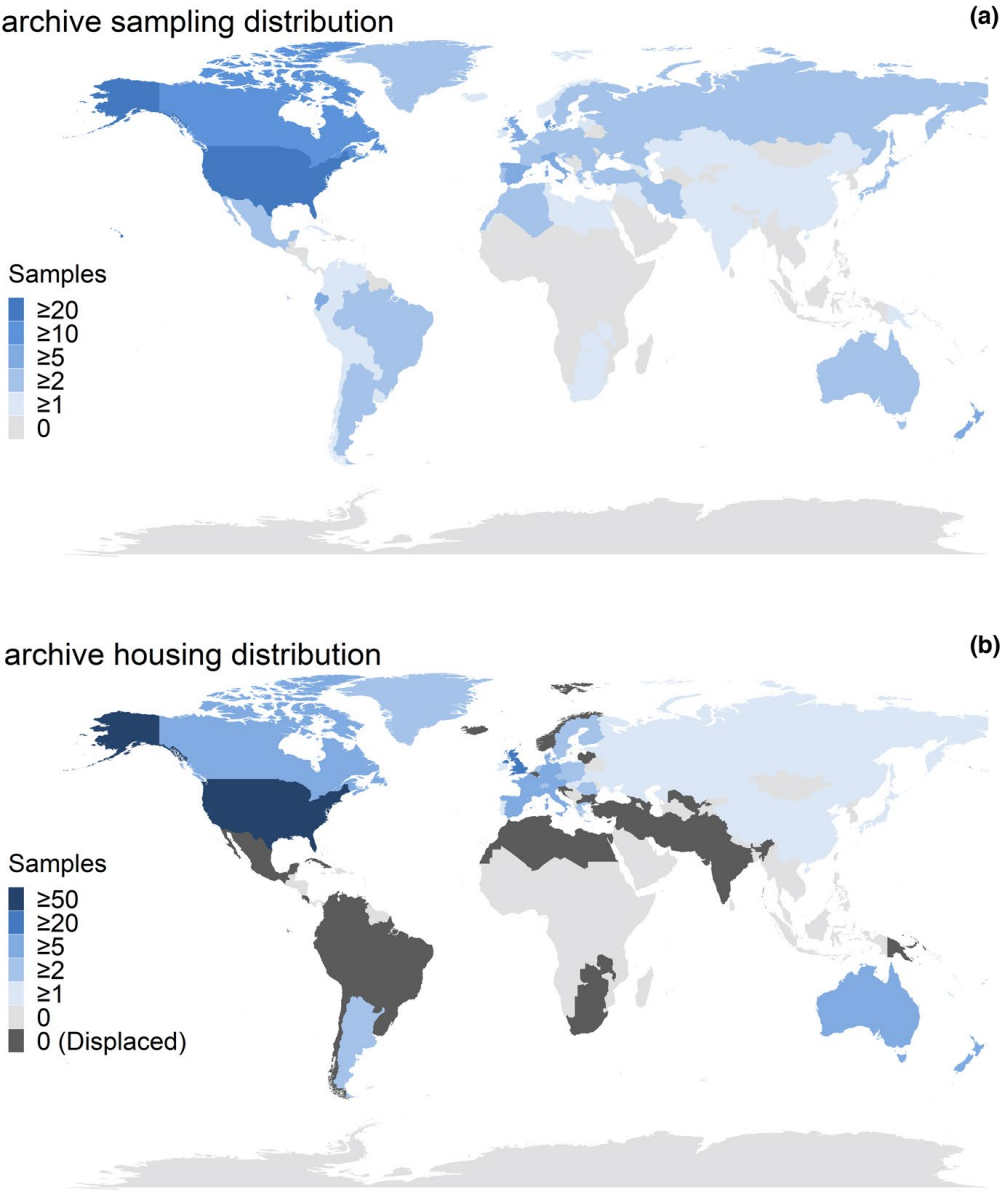
Distribution of countries sampled for temporal genomics studies. (a) Countries in which museum samples originated. Countries were only counted once per study even if multiple locations, populations, time points, or species were sampled. (b) Countries in which archived samples were housed. Countries were only counted once per study even if multiple locations, populations, time points, or species were sampled. Countries where samples were originally collected but housed elsewhere are labelled as “Displaced” in (b). Studies that do not use museum samples are not included in the sample counts.

Our literature search also revealed temporal genomics studies from a wide, albeit biased, geographical distribution. While many museum or archival samples originated in western Europe (e.g., France, Demark, UK, Italy, Spain, etc.; 27%), the USA (16%), or Canada (5%), there were a substantial number of studies across the globe, with over 70 countries represented in our database (Figure 2a). However, when we looked at the host country or museum where the historical samples were housed, the distribution was much more skewed: archived samples came from only 32 countries, with 77% of these collections found in either western Europe (44%), the USA (29%), or Canada (4%; Figure 2b). This discrepancy in origination vs. sampling location for many of the samples highlights the legacy of colonialism that continues to structure temporal genomics studies and other research based on museum specimens (Marks et al., 2021). Many samples currently housed in western institutions were originally collected during research expeditions to stolen territories or land (Trisos et al., 2021). Furthermore, of the 43 studies in our data set with museum samples that originated outside the USA, Canada, or western Europe, fewer than half had an author affiliated with an institution from the country of sample origin that was not Australia or New Zealand (Figure S1). The often complicated and painful history of museum collections is a poignant reminder that careful consideration of access, equity and inclusion remains crucial when it comes to ethically conducting museum‐based work. While the number of collaborative genomics projects, such as the African BioGenome Project and DIPnet, has been steadily increasing over the years and global museum databases make accessing archival data easier than ever before (Lewin et al., 2022), much work has yet to be done. Just as the field has progressed with better laboratory techniques, so too must it progress with equitable standards that empower host country institutions by codesigning research, supporting accessible scientific infrastructure, building long‐term collaborations and fostering the development of local analytical expertise (Asase et al., 2021; Prendergast & Sawchuk, 2018; Stefanoudis et al., 2021).

### 2.2 Drivers of temporal genomics change

**FIGURE 3:**
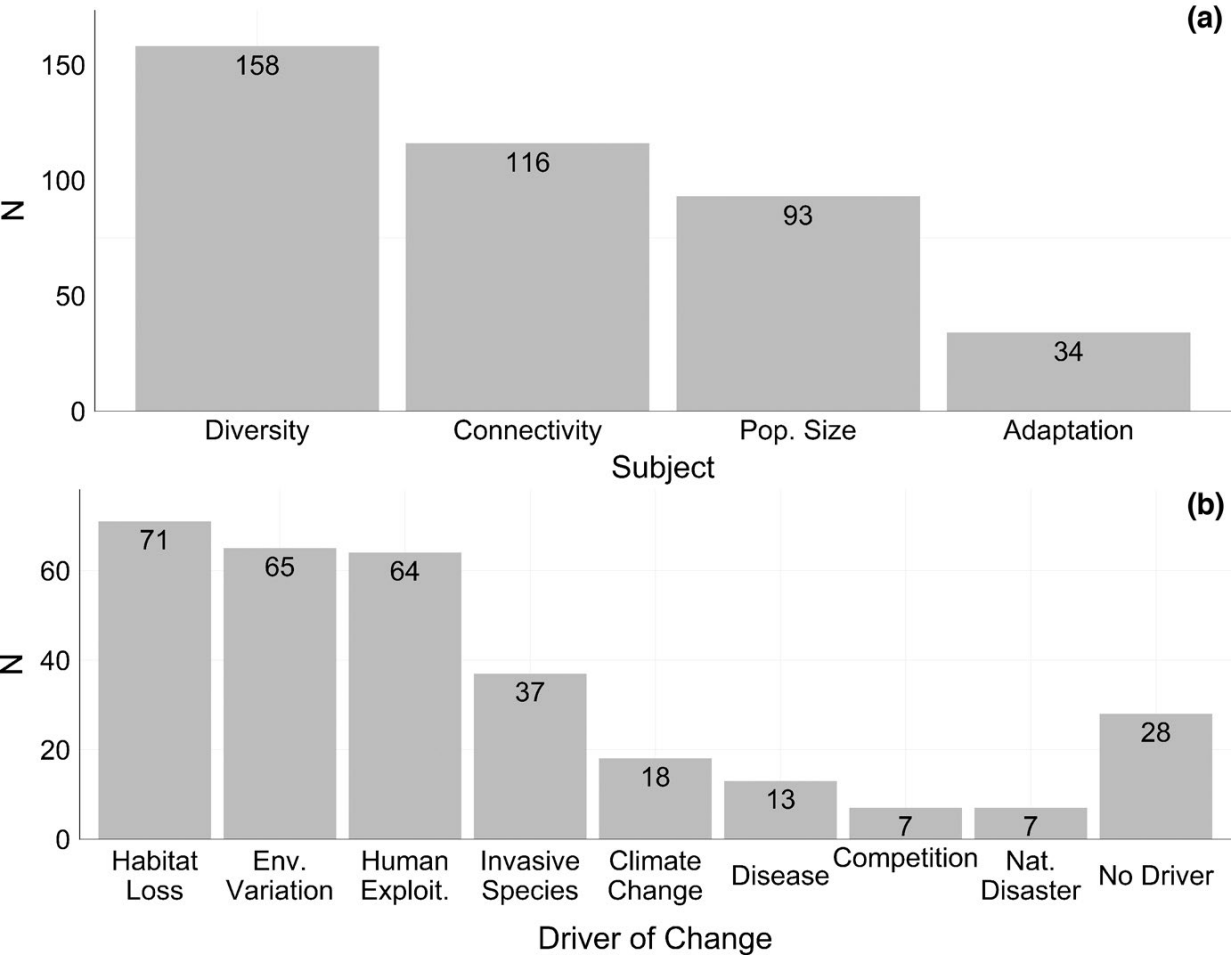
Distribution of temporal genomics studies across subject (a) and driver of change (b). Numbers within bars represent counts per subject/driver of change. Studies could be assigned more than one subject and/or driver. Studies where a driver of change could not be identified were assigned “No Driver”.

Using the entire data set of 218 studies, we sought to identify shared motivations behind temporal genomics studies. Studies investigating temporal changes in genetic diversity were most frequent, comprising 39% of our data set, followed by those investigating changes in population structure or connectivity regimes (29%), population size or demographic histories (23%) and signatures of adaptation (9%; Figure 3a). The recent availability and declining cost of sub‐ and whole‐genome sequencing (WGS) have enabled more effective scans for selection and we expect that the number of adaptation studies will grow rapidly in the coming years.

In general, we found that most temporal genomic studies in our data set resulted from long‐term genetic monitoring programmes that assessed periodic changes in population sizes (Osborne et al., 2012) and evaluated the success of captive breeding strategies (Jensen et al., 2018), or relied on archival data sets to determine the evolutionary consequences of well‐known population declines (Bergner et al., 2016). Very few studies in our data set were designed with the central goal of evaluating fundamental questions in evolutionary biology. However, one exception by García‐Navas et al. (2015) explicitly tested how selection and gene flow combined to counteract genetic drift and maintain high genetic diversity in fluctuating snow vole populations, finding that density‐dependent dispersal helped offset drift by bringing in novel alleles. Because a key advantage of temporal genomics is the ability to view “evolution in action,” substantial opportunities exist to test evolutionary theory with temporal replicates and to identify how evolutionary forces and the underlying genomic architecture combine to shape population dynamics. Genomic time series may be particularly useful for identifying mechanisms of local adaptation, characterizing the process of hybridization and establishing feedback loops between population dynamics and evolutionary processes. As such, the continuation of both applied and fundamental research will be pivotal to the continued growth of evolutionary biology.

Furthermore, for each study, we identified whether the authors were investigating genetic change due to specific anthropogenic or natural factors. We found that most studies focused on change due to habitat loss (23%), human exploitation (21%), environmental variation (21%), or invasive species (12%; Figure 3b). Many studies tracked change due to more than one anthropogenic or natural force (e.g., diversity loss as a result of both exploitation and habitat loss). Interestingly, we found a scarcity of studies investigating how extreme events facilitate rapid genetic change, such as from sudden disease outbreaks (Lilley et al., 2020) or natural disasters (Hsu et al., 2017). Since such events are largely unpredictable, studies on these events would benefit from the maintenance of long‐term genomic monitoring programmes. Finally, the most common driver of genetic change varied by taxon (Figure S2). While environmental variation was the most prevalent driver for many taxa, habitat loss and/or human exploitation were the most common for mammals and birds. In addition, most climate change‐focused studies tracked genetic change in longer‐lived organisms.

### 2.3 Study design trends in temporal genomics studies

**FIGURE 4:**
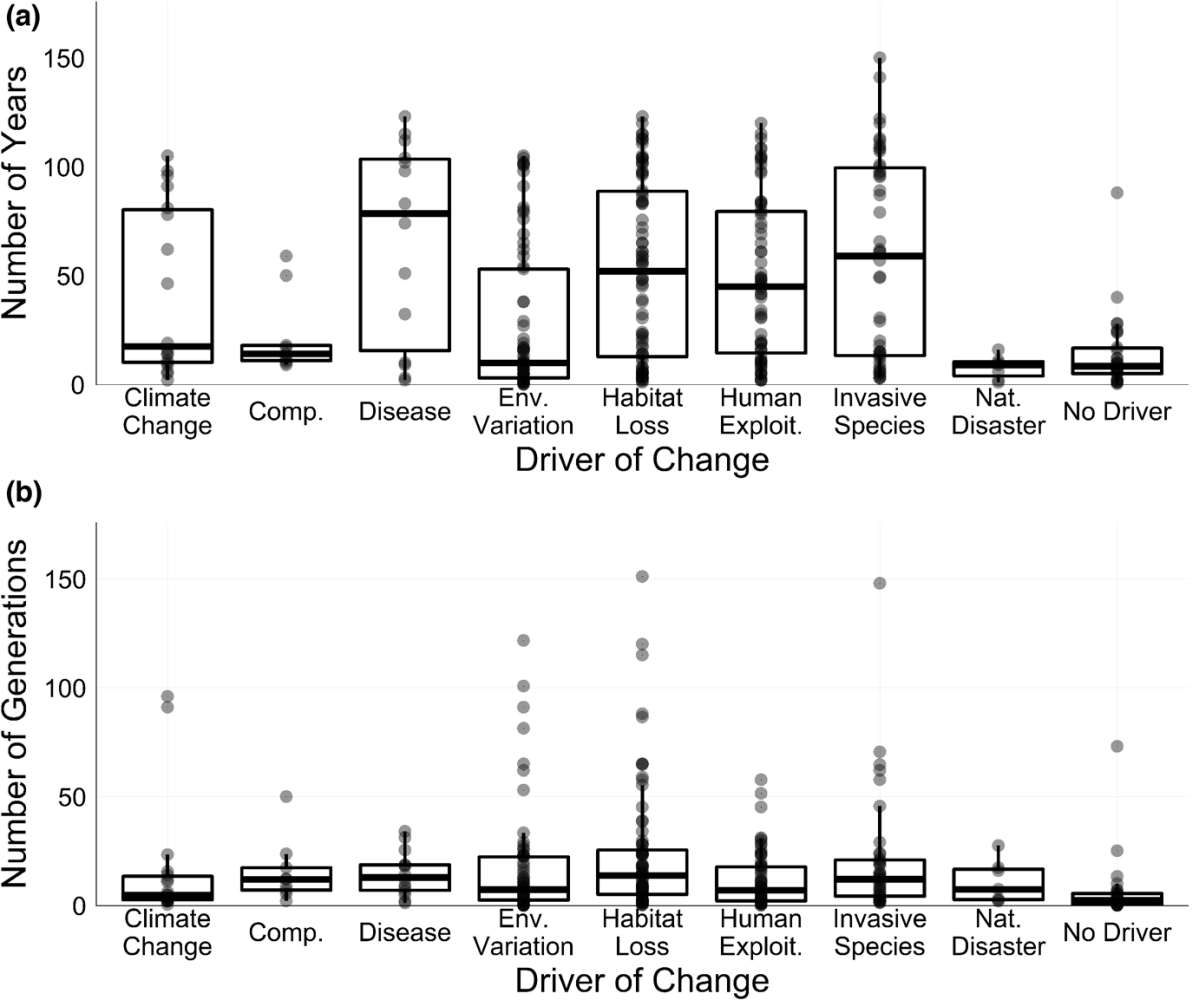
Maximum time span over which genetic change was assessed either in years (a) or generations (b). Points represent unique sampling schemes in individual studies. Studies could be assigned more than one driver. Studies where a driver of change could not be identified were assigned “No Driver.” For (b), six sampling schemes spanned more generations than are shown on the *y*‐axis. These are: Env. Variation studies spanning 167 and 347 generations, a Habitat Loss study spanning 750 generations, and Invasive Species studies spanning 200, 220 and 301 generations.

As study design is critical to statistical and analytical power, we examined which sample designs and genetic markers were most frequently used in our data set. When we reviewed common temporal sampling regimes, we found that studies included an average of three unique time points (or “periods” when temporal samples were pooled across several years). In total, 104 studies (48%) looked at change across only two time points, often divided into “before” and “after” some event of interest (Figure S3). Twenty‐one studies (10%) observed change over three time points, while 13 (6%) included over 10 time points in their analyses. Forty‐one studies (19%) included multiple temporal sampling regimes (e.g., more temporal replicates were available for only certain populations or time points were pooled together for certain analyses). Study length was a median of 17 years, or eight generations. However, this varied widely depending on the driver of interest (Figure 4). We also found that exactly half of all studies (50%) utilized museum or archival specimens (“opportunistic studies”), while the other half (50%) analysed samples entirely collected by the study authors or as part of a long‐term monitoring plan (“predesigned studies,” see Figure S4 for a map of their global distribution).

**FIGURE 5:**
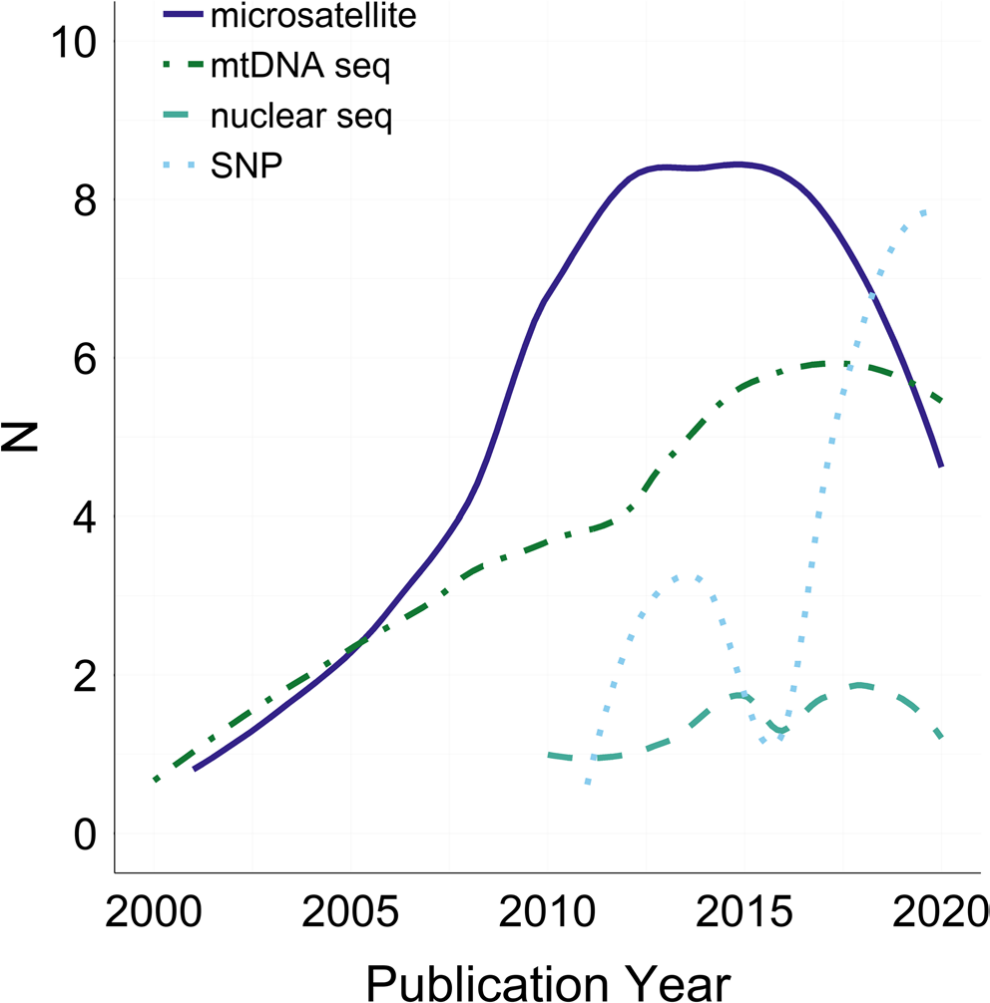
Trends in marker usage over time. HRM and X‐chromosome markers do not show on the figure as there was only one study for each (HRM = 2017; X‐chromosome = 2015). HRM, high‐resolution melt.

While most studies investigated change using microsatellites (47%) or mitochondrial DNA sequences (34%), we found that single nucleotide polymorphisms (SNPs) increased in prevalence in recent years and are currently the most widely used marker (Figure 5). Similarly, while the majority of studies relied on Sanger sequencing, next‐generation sequencing platforms such as Illumina and Ion Torrent that facilitate whole‐genome and reduced‐representation sequencing (e.g., RAD and sequence‐capture) have become more common as well. As sequencing costs continue to fall, the creation of annotated reference genomes for additional nonmodel organisms will expand the types of questions the field can explore temporally in the future. The use of WGS and short‐ or long‐read data to identify structural variants (e.g., inversions, translocations, copy number variants) will advance our understanding of the mechanisms underlying adaptation and hybridization (Mahmoud et al., 2019). Whole‐genome resequencing also enables the detection of runs of homozygosity in bottlenecked or inbred populations, which can help refine our perception of how diversity is lost over time (Ceballos et al., 2018).

## 3 BEST PRACTICES

Temporally spaced samples offer a unique opportunity to directly observe the genomic composition of populations at different time points and document the pace and trajectory of microevolution through time. Yet, researchers often face unique study design challenges when investigating genomic changes using temporal samples. A common example is the limitation of temporal and spatial samples in museum collections and other natural history archives. The general condition of samples and preservation methods can also impact the quality and quantity of genomic material, which can subsequently dampen sequencing output. Additionally, project costs force a tradeoff between the number of individuals, markers and time points that can be sequenced and species‐specific traits (e.g., generation time, population size) can pose unique limitations on study design. Furthermore, half of the studies we reviewed employed predesigned sampling, indicating a need for sampling design recommendations for planned temporal genomics studies as well. Here, we discuss practical considerations when designing temporal genomics studies with a specific focus on analyses that investigate change in diversity, adaptation, connectivity and population size (Boxes 1, 2, 3, 4). These recommendations are written with scientific study design in mind; we invite readers interested in temporal genomics from a monitoring perspective to refer to Hoban et al. (2021) and Hoban et al. (2022) for conservation‐focused guidance.

BOX 1 **Estimating changes in genetic diversity using temporal genomic data sets**

**Table:** 

Data type	Metric, software, or analysis	Recommendations
mtDNA	Standard genetic diversity metrics such as haplotype diversity, *H*_E_/*H*_O_, and nucleotide diversity (π) with software such as arlequin and genepop.	Useful for investigating genomic change over shorter time periods, though demographic signals dissipate more quickly. Sample as many individuals as possible.
SNPs and nuclear sequence data (reduced‐representation sequencing, WGS)	Standard genetic diversity metrics such as expected and observed heterozygosity (*H*_E_/*H*_O_), allelic richness, temporal changes in allele frequencies, and π with software such as pixy and the R package adegenet. When sequence data are available, changes in SFS, runs of homozygosity to estimate levels of inbreeding and genomic load (as the ratio of deleterious to synonymous variants) can also be calculated, with software such as angsd, rohan and generode.	Reduced‐representation sequencing and WGS are best to retain useful sequence information. Consider sequencing to higher depth to avoid confounding rare variation with sequencing error in historical samples. Make sure to establish appropriate historical baselines to avoid underestimating diversity loss. If needed, rarify allelic richness and bootstrap to estimate confidence intervals.

Assessing historical changes in genetic variation is one of the most pervasive goals in temporal genomics, as the loss of genetic diversity can have serious ramifications for adaptation and population persistence. The preservation of genetic diversity has also been included as a post‐2020 goal in the Convention on Biological Diversity's global biodiversity framework (Hoban et al., 2023). As scientific study design, not conservation, is the focus of this review, we direct researchers interested in genetic monitoring to Hoban et al. (2021, 2022) and references within for a more rigorous overview of genetic diversity from a management perspective. However, diversity can be measured in myriad ways and is of interest beyond its intrinsic conservation value.

Different metrics of diversity provide distinct types of information. As an example, richness metrics, such as allelic richness, are particularly sensitive to demographic events because rare alleles are quickly lost following a population decline (Nei et al., 1975). Evenness metrics, such as heterozygosity, are lost at a slower rate after a bottleneck. Because of this nonlinear nature of allelic loss (Lacy, 1987), temporal genetic diversity analyses are particularly sensitive to baseline estimates. Good baselines (e.g., temporal samples that occur at or before the event of interest) are key to accurately characterizing diversity change. Incorrect baselines can lead to an underestimation of diversity loss (see Hartmann et al., 2014). Finally, temporal changes in genetic load (the presence of deleterious variants) can help detect the degree to which a post‐bottleneck population has either accumulated maladaptive mutations or purged them via purifying selection (Díez‐del‐Molino et al., 2018; Kutschera et al., 2022).

Marker choice can also greatly influence both genetic diversity estimates and the pattern of diversity loss or gain observed through time. On their own, SNPs tend to inform a relatively limited range of diversity metrics. For example, while average pairwise genetic distance (π) can be evaluated using only SNPs, without complementary sequence data, issues with missing data and a lack of monomorphic sites can complicate interpretation (Korunes & Samuk, 2021). When sequence data are available (either with WGS or reduced‐representation sequencing), haplotype reconstruction and the inclusion of monomorphic sites provide valuable additional information (Leitwein et al., 2020). Sequence data can be used to estimate levels of inbreeding by measuring runs of homozygosity (Renaud et al., 2019) and phased haplotypes can be treated as unique loci to quantify allelic richness. However, caution should be taken when using low‐coverage WGS data to estimate temporal changes in genetic diversity, as it can be difficult to differentiate sequencing errors from rare genomic variants (Kousanthanas et al., 2017; Lou et al., 2021). Given that low‐frequency alleles are often the first to be lost during genetic bottlenecks, programs that account for such issues should be used when possible (e.g., upstream base quality recalibration: Ni & Stoneking, 2016; downstream SFS estimation: Nielsen et al., 2012).

Finally, compared to nuclear DNA, mitochondrial DNA (mtDNA) data are easy to obtain and can provide valuable insight into temporal changes in genetic diversity, particularly as mtDNA is present in much higher quantities and thus may be more easily extracted from degraded historical samples. However, as mtDNA is inherited maternally, it has an effective population size that is a quarter that of nuclear DNA. This means that the time to realized change in diversity after a population bottleneck is shorter in mtDNA than in nuclear DNA, which can be useful but can also erase population history more quickly (Pfau et al., 2019).

BOX 2 **Identifying adaptation using temporal genomic data sets**

**Table:** 

Data type	Metric, software, or analysis	Recommendations
SNPs	Identify regions of the genome with large allele frequency changes as putatively under selection. Detect covariation of many loci through time as an indication of polygenic selection (see Buffalo & Coop, 2020; Foll et al., 2015; Gompert, 2016; Malaspinas, 2016; Schraiber et al., 2016 for analysis methods).	WGS (including low‐coverage) is best for detecting selection across the genome. Larger sample sizes are better for detecting loci of smaller effect. If sample size is small, consider running power analyses.

Sampling genomes from multiple time points offers the chance to study both the timeline and the speed of selection. One approach to measure genomic changes underlying evolution in real time over multiple generations are “evolve and resequence” experiments, which combine experimental evolution in controlled laboratory or field mesocosm settings with next‐generation sequencing to infer selection. However, experiments under these controlled conditions pose challenges for generalizing the resulting selection dynamics (Dehasque et al., 2020). Using temporal samples of wild, sexually reproducing populations provides a unique opportunity to infer selection by measuring changes in allele frequency while concurrently considering ancestral demography. When possible, sampling should be conducted before and after a new selective pressure, such as an environmental change or novel anthropogenic stressor, is introduced. If a study is opportunistic, museum collections or natural history archives may have specimens that were collected before a new selective pressure was introduced (see Therkildsen et al., 2013). Samples across multiple generations are needed to fully detect signatures of selection.

Marker choice is key for studies focusing on adaptation, as this determines the fraction of the genome scanned for selection. Studies only genotyping fractions of the genome (e.g., RADseq and microsatellite studies) can detect selection at genotyped loci and linked portions of the genome, but WGS is needed to detect selection across the remainder of the genome (Lilley et al., 2020; Pinsky et al., 2021). Low‐coverage sequencing can be used to obtain whole genomes at a cost comparable to reduced‐representation sequencing (Gignoux‐Wolfsohn et al., 2021; Lou et al., 2021). Analytical techniques have been developed specifically to detect selection from changes in allele frequencies and calculate selection coefficients from temporal samples, such as wfabc (Foll et al., 2015) and related methods (Gompert, 2016; Malaspinas, 2016; Schraiber et al., 2016). Other temporal methods can detect polygenic selection from the covariance in allele frequency changes through time (Buffalo & Coop, 2020).

Sample size determines the smallest allele frequency that can be quantified, and therefore, the smallest selection coefficient that can be detected (see simulations in Pinsky et al., 2021). Small sample sizes can only identify loci of large effect, such that most polygenic selection is not detectable (see Yeaman et al., 2016 for examples of detecting polygenic selection). Thus, we suggest sequencing as many individuals per time point as possible. However, most adaptation studies reviewed here still reported having enough statistical power to identify outlier loci with relatively small sample sizes (e.g., ~30 samples per time point worked well for Gignoux‐Wolfsohn et al., 2021, and 40–50 for Therkildsen et al., 2019). When low sample sizes are all that is available, we recommend analysing temporal variation in allele frequencies with the Bayesian method detailed in Sandoval‐Castellanos (2010), as it performed well in small populations with many alleles.

BOX 3 **Estimating changes in connectivity using temporal genomic data sets**

**Table:** 

Data type	Metric, software, or analysis	Recommendations
Microsatellites or SNPs	Genetic differentiation metrics (e.g., *F*_ST_, *G*_ST_, *D*) to quantify the level of genetic dissimilarity between populations.	Sample multiple individuals at populations across the study area and sample the same locations across time points. If fewer markers (e.g., microsatellites), more individuals may need to be sampled to increase estimate precision. Perform power analysis if sample sizes are too low (Garcia et al., 2020).
	Population assignment methods (e.g., structure, instruct, admixture, dapc) to quantify population structure and admixture.	Sample multiple individuals across the study area and through time. Consider the time needed for the populations to reach equilibrium.
	Parentage analyses to directly quantify dispersal between natal sites and observed location.	Sample consecutive generations. Microsatellites or a few hundred SNPs are usually sufficient (Hauser et al., 2021).

To understand temporal changes in spatial genetic structure, the balance between sequencing more individuals or more loci can be particularly challenging, as researchers must balance sampling effort across both space and time. Sampling multiple individuals at populations across the study area and considering the lag between changes in connectivity and our ability to detect these changes is important (Landguth et al., 2010). Compared to selection scans, gene flow studies require fewer samples and less of the genome sequenced, as each temporal replicate provides an additional estimate of connectivity.

Genetic differentiation methods utilize putatively neutral genetic markers to estimate the level of genetic similarity between populations and provide an estimate of gene flow. Estimates of genetic differentiation that account for multiple alleles (e.g., *D*, *G*'_ST_, *G*″_ST_) may be better for highly polymorphic loci (e.g., microsatellites) because other estimates are down‐biased (Meirmans & Hedrick, 2011). Similarly, it may take more generations after the introduction of a connectivity barrier to detect change with *F*_ST_ estimators compared to the shared alleles' statistic (Dps; Landguth et al., 2010). Finally, novel approaches using graph theory and network models are being developed that specifically incorporate time into spatiotemporal patterns of connectivity (Draheim et al., 2018; Fenderson et al., 2020).

Parentage analyses use temporal sampling to identify parent–offspring relationships (Catalano et al., 2021) and directly infer dispersal. With these types of analyses, both population size and sampling effort directly impact the number of parent–offspring relationships that can be detected (Hauser et al., 2021; Saenz‐Agudelo et al., 2009). Clustering approaches (e.g., structure, dapc) partition genetic variation into genetically distinct groups. These types of techniques can be used to evaluate if allele frequencies have changed through time (isolation‐by‐time) by including all time points and assessing whether time periods cluster together (Wolf et al., 2012).

BOX 4 **Estimating changes in population size using temporal genomic data sets**

**Table:** 

Data type	Metric, software, or analysis	Recommendations
Genotypic data (microsatellites or SNPs)	Multigenerational genetic pedigrees to estimate *N*_e_, the number of breeding individuals, and reproductive success (colony2). Observations in capture–mark–recapture analysis to estimate *N*_c_, detection probability, and apparent survival (mark). Changes or stability of *N*_e_ by assessing excess linkage disequilibrium and excess heterozygosity (ldne/neestimator). Estimate average size over time based on allele frequency change (mlne).	Sample a substantial proportion of the population. Larger marker sets, including low‐coverage WGS, allow researchers to detect more distant familial relationships, including cousins (Waples et al., 2019). Sample at least 1% of the census population when using single‐sample estimators such as excess linkage disequilibrium analysis. Sample enough individuals to accurately estimate allele frequencies; sample multiple generations apart.
Site frequency spectrum (SFS) from biallelic SNPs	SFS‐derived summary statistics. Detect recent *N*_e_ changes using contemporary data (fastsimcoal2, momi2).	Sample large numbers of individuals (>100) for SFS‐summary statistic. Smaller sample sizes (>15) might be adequate with >25,000 loci (Nunziata & Weisrock, 2018).

When designing studies aimed at quantifying population size using temporal genomic data, extensive sampling is often important, and the best sample sizes will frequently be determined by the analytical methodology to be employed. A first class of methods uses unique genotypes collected over multiple years (often generated via noninvasive sampling) in a probabilistic framework, such as the capture–mark–recapture framework implemented in program mark (White & Burnham, 1999) or in frameworks designed specifically for estimating population size using noninvasive samples such as capwire (Miller et al., 2005). These studies focus on census population size (*N*_c_) and can often co‐estimate other parameters (including detection probability and apparent survival) relevant to population dynamics. Moderate numbers (15 or more; McKelvey & Schwartz, 2004) of highly polymorphic markers such as microsatellites or larger numbers of SNPs (hundreds or more, Waples et al., 2019) are usually sufficient for accurate identification of individuals and closely related individuals. These methods work best when capture probabilities are high (Lukacs & Burnham, 2005) and the size of the study population is small (Miller et al., 2005).

Other methods focus on estimating the effective population size (*N*_e_), which determines the rate of inbreeding and genetic drift (Nei et al., 1975). Several methods have been developed for estimating *N*_e_ from single samples of multilocus genetic data based on assessing excess linkage disequilibrium (Waples & Do, 2008), excess heterozygosity (Zhdanova & Pudovkin, 2008), or sibship frequency (Wang et al., 2016). Sibship‐based estimation appears particularly robust to factors that can bias other methods such as nonrandom mating and population subdivision (Wang et al., 2016). Single‐sample estimators often require sampling a substantial proportion of the population to accurately estimate population size (about 1%, Marandel et al., 2019).

A final class of models uses allele frequencies to infer *N*_e_ and changes in *N*_e_. The SFS, which can be derived from SNP or WGS data, contains extensive information on changes in *N*_e_ over time. The SFS can be used to infer past demography using either approximate Bayesian computation or model‐based methods (e.g., Excoffier et al., 2021; Kamm et al., 2020). Another allele frequency‐based method infers the harmonic mean of *N*_e_ over a particular time period based on allele frequency change over that time period (Jorde & Ryman, 1995). An extension of this allele frequency change method is implemented in the program mlne (Wang, 2022), which has been shown to be particularly useful for estimating *N*_e_ in the presence of migration (Gilbert & Whitlock, 2015). To detect recent changes using only contemporary data, SFS methods require large sample sizes (>100 individuals; Beichman et al., 2018), as accurately quantifying the frequency of rare alleles is often necessary for inferring recent population changes. A simulation study using temporal data with an SFS method found that smaller sample sizes (>15 individuals per time point) were adequate when many SNPs (25,000–50,000) were genotyped (Nunziata & Weisrock, 2018); however, other simulation studies have shown that larger sample sizes provide higher power to detect declines and more accurate inferences of *N*_e_ with temporal data, particularly when historical population sizes were large (Reid & Pinsky, 2022).

Biological considerations are also important. For example, the strength of the signal of genetic drift depends on the number of generations that have elapsed between sampling points (Wang et al., 2016). Population structure can also affect the sample size needed with these methods. For instance, estimating *N*_e_ for species with expansive populations and long‐distance dispersal may be challenging because it is difficult to sample a large enough proportion of the population (Marandel et al., 2019). Even in panmictic species (e.g., many marine fish) where a single well‐mixed population is expected, “sweepstakes” breeding events and aggregations of related individuals can cause deviations from this expectation that could potentially bias inferences of *N*_e_ and genetic diversity over time (Broquet et al., 2013).

### 3.1 Temporal sampling design

In many cases, researchers will be limited by temporal sample availability, particularly when sourcing from archival data sets. However, in this section we emphasize other temporal sampling schemes as well to provide guidance for predesigned studies and cases where sample availability is not a limitation.

**FIGURE 6:**
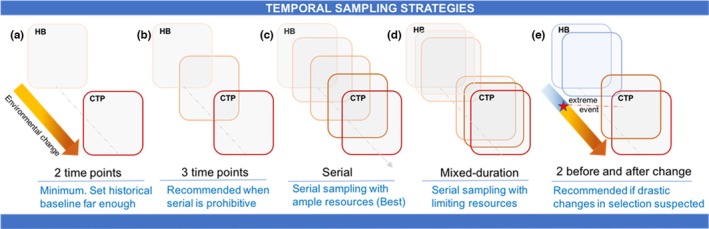
Sampling strategies for temporal genomic studies. Boxes represent sampled populations at a given environmental state (depicted by colour) in time. All temporal genomics studies have at least two time points (a), while three is recommended when feasible (b), and (c) serial sampling (more than three time points) is optimal. When studying linked selection, Buffalo and Coop (2019) recommend (d) a mixed‐duration scheme as a compromise to prolonged serial sampling if resources are limited; and (e) sampling at least two temporal samples before and after extreme events that substantially change selective pressures and allele frequencies. CTP, contemporary time point; HB, historical baseline.

By design, temporal studies require at least two time points (Figure 6) and the oldest becomes a historical baseline for comparison. Setting this historical baseline far enough back in time is essential to accurately capture evolutionary events (Jensen & Leigh, 2022). For example, when estimating changes in genetic diversity following a population bottleneck, obtaining samples prior to the event is crucial, as genetic drift expedites the loss of rare alleles (Lacy, 1987). Failure to properly sample the population before the bottleneck could result in an underestimation of diversity loss. When working with archival data, collection biases (Meineke & Daru, 2021) and missing metadata may further complicate the identification of an appropriate historical replicate for contemporary populations. Life history traits can help guide researchers in both determining appropriate historical baselines and predicting expected rates of evolutionary change. Species with higher *N*_e_ values are more likely to lose rare alleles at a slower rate, while systems that experience high gene flow may experience more transient diversity loss and faster recovery (Bernard et al., 2016).

While insightful inference can be reached with a well‐established baseline and only one additional time point, additional knowledge can be gained by adding more time points, when possible, to track the trajectory of change over time (Figure 6b). Compared to only two time points, adding samples from intermediate time points improves the statistical power of analyses and can reveal the dominance of alleles under selection (Foll et al., 2015) and polygenic selection (Buffalo & Coop, 2020). Sampling populations serially (here defined as having more than three time points, Figure 6c) provides the widest array of potential analyses while increasing the resolution, power and ability to identify sampling noise (Buffalo & Coop, 2020; Foll et al., 2015; Gauthier et al., 2020). However, despite its myriad benefits, serial temporal sampling remains difficult in many cases due to low sample availability and budget restrictions. While decreasing sequencing costs are likely to increase the number of temporal samples in future studies, researchers can design alternative serial sampling schemes if available resources are limited. For example, Buffalo and Coop (2019) suggest a “mixed‐duration sampling design” (Figure 6d) as a compromise to prolonged serial sampling when trying to identify signatures of rapid adaptation. With this design, researchers sample every generation for a few generations, skip several generations and then begin again (sampling generations 1–4, 11–14, etc.). This type of sampling is particularly effective for detecting polygenic selection because the variance in allele frequency change across loci in adjacent generations is only affected by heritable variation in offspring number, while temporally distant generations also accumulate temporal autocovariance in neutral alleles due to linked selection.

Finally, the optimal number of temporal replicates is often influenced by species' life history traits, the type of evolutionary change being observed and the driver of interest. For instance, as evolutionary change accumulates over generations, it is important for researchers to consider the number of generations that have elapsed between sampling points when setting expectations. If long generation times make historical samples inaccessible, sampling across cohorts or age groups in contemporary populations can be a useful proxy for different time points, particularly if individuals can be accurately aged (Schmidt et al., 2018). Moreover, when investigating fluctuating selection, as might occur across seasons for species with multiple generations per year, at least two temporal samples should be collected per season (Buffalo & Coop, 2019; Figure 6e). Both fluctuating selection and sampling noise shared between adjacent time points can generate negative covariance in allele frequency change over time, making it difficult to differentiate these two sources of frequency change (Buffalo & Coop, 2019). However, by comparing time points both within and across seasons (or across other periods of selective change), the sampling noise shared between adjacent time points can be estimated separately from the impact of linked selection on allele frequency variation over time (Buffalo & Coop, 2019).

### 3.2 Marker choice, sample sizes and analyses

Marker choice and sample size are also crucial considerations when designing temporal studies and can greatly impact both statistical power and inferential ability. In general, larger numbers of both samples and loci allow for a greater array of analyses and offer higher resolution and confidence. As such, most temporal genomics studies now use SNPs (Figure 5). With these next‐generation methods, a tradeoff exists between sequencing the entire genome or a reduced portion of it with targeted‐capture, restriction‐site‐associated (RAD) loci, exome, or related sequencing approaches. Although sequencing smaller fractions of the genome has traditionally been cheaper, this may not always be the case when dealing with temporal samples. For example, hDNA is often too degraded for reduced‐representation sequencing of RAD loci. Despite these limitations, reduced‐representation sequencing of historical samples has proven successful in the past (Bi et al., 2013). Furthermore, recent advancements in capture‐based protocols for hDNA have shown that such methods are cost‐effective and can yield high‐quality downstream data (Suchan et al., 2022), particularly when paired with bioinformatics pipelines designed specifically for historical capture‐based data (Gauthier et al., 2020).

When reduced‐representation library preparation proves too costly or ineffective, low‐coverage WGS provides a useful alternative and has also become increasingly prevalent in temporal genomics studies (Gignoux‐Wolfsohn et al., 2021; Pinsky et al., 2021). Low‐coverage sequencing does not come without its own challenges, however, as it often yields read depths that are too low to call genotypes with certainty (Lou et al., 2021). This uncertainty forces researchers to compute genotype likelihoods and constrains downstream analyses to those that either incorporate genotype uncertainty into probabilistic frameworks, or focus on genome‐wide patterns and summary statistics (e.g., site frequency spectra (SFS) or linkage patterns; Lou et al., 2021). Boxes 1 and 2 highlight some of the unique challenges genotype likelihoods pose for population genomics analyses (Boxes 1 and 2).

When study budgets force a tradeoff between sequencing more individuals or loci, analysis types can help determine which to prioritize. For instance, large sample sizes are recommended for studies seeking to detect rare immigrants or recent changes in *N*_e_ (where rare alleles are particularly informative; Box 4). In contrast, sequencing large fractions of the genome is important if low numbers of individuals per time point are unavoidable or when detecting adaptive or structural variation is important (Lou et al., 2021). Finally, maintaining similar sample sizes across time points may be a priority when uneven sample sizes can affect downstream analyses (e.g., larger contemporary sample sizes upwardly biasing allelic richness estimates). Boxes 1, 2, 3, 4 provide guidelines and recommendations aimed at assisting researchers designing temporal studies focused on changes in genetic diversity, adaptation, connectivity and population size, respectively.

Ultimately, the appropriate sample size and marker choice will vary depending on the question, the type of markers and genomic resources used and the samples available from the study system. Simulations can be useful for evaluating the statistical power of a particular combination of temporal sampling scheme and analytical method to discriminate between alternative hypotheses (Nunziata & Weisrock, 2018; Reid & Pinsky, 2022). While the potentially large number of input parameters for simulations (e.g., mutation rate, chromosomal organization and recombination rates that can vary across the genome) may initially seem daunting, libraries of standardized population simulations now exist (Lauterbur et al., 2022) for conducting realistic simulations in both model and nonmodel species. These resources can provide useful starting points for researchers interested in designing their own simulations.

### 3.3 Common pitfalls in temporal genomics studies

Many temporal genomics studies use samples with poor DNA preservation and researchers should pay particular attention to the technical shortcomings associated with historical samples. Historical reads are often shorter, contain DNA damage and have higher sequencing error rates, which can reduce sequencing efficacy and generate byproducts that can be mistaken for, or mask, temporal genomic change (e.g., filtering out minor alleles to remove sequencing errors can also inadvertently remove informative rare variants). Targeted‐capture sequencing and sequencing to higher depth can help overcome some of these issues, as can incorporating bioinformatics programs designed to address DNA degradation and contamination (e.g., fastq screen to remove exogenous DNA: Wingett & Andrews, 2018; mapdamage to detect damage patterns: Ginolhac et al., 2011). In addition, reference genomes are typically constructed from modern individuals, which introduces the potential for reference mapping biases, wherein sample reads that differ from the reference do not map properly, leading to false variant calling. As a population evolves through time and accumulates new mutations, it begins to diverge from previous generations. Thus, populations that were sampled many generations apart may be different enough at the genomic level to cause mapping difficulties, particularly if the reference genome was constructed using individuals from only one time point. These mapping biases may be further amplified by the typically shorter reads produced by historical samples. Utilizing mapping strategies created specifically for ancient (aDNA) or hDNA data can help combat some of these issues (Oliva et al., 2021).

Another common challenge observed in temporal genomics studies focusing specifically on adaptation stems from genomic markers that only represent part of the genome. Studies, after all, can only test for selection in the portion of the genome that is genotyped or linked to genotyped loci. While strong signals of selection can be detected with reduced‐representation sequencing, including genotype–environment associations across space (Caputi et al., 2019), a lack of signal leaves open the possibility that selection is acting on other parts of the genome. WGS can help avoid this issue (e.g., Gignoux‐Wolfsohn et al., 2021; Lilley et al., 2020), though most WGS studies do not address structural and other forms of variation beyond SNPs (but see Catanach et al., 2019). When WGS is not feasible, capture‐based sequencing or SNP microarrays that target previously identified candidates of selection may be appropriate (e.g., Czorlich et al., 2022).

Finally, interpreting observed genetic changes should also be done with caution when populations are small or structured, given that alleles drift more easily in these than in large populations. As populations can be replaced or move, researchers need to test whether they are sampling the same cohesive genetic deme across multiple time points (Box 3). One way to account for such replacement issues is to utilize methods that identify temporal population relationships and assess the ancestral history between modern and historical populations (Schraiber, 2018).

## 4 CONCLUSION

### 4.1 Emerging opportunities for temporal genomics

Temporal genomics constitutes a uniquely useful framework for directly investigating changes in the genomic composition of wild populations over time and the field of temporal genomics has already greatly expanded our understanding of how recent environmental changes have impacted population sizes and connectivity. Although our review identified adaptive change as currently the least‐studied aspect of temporal genomics, the increasing feasibility of whole‐genome resequencing will enable significant leaps in the accessibility of recent adaptation as a field of study in the near future. While studies using only contemporary samples can identify outlier loci and environmentally associated loci of large effect, temporal genomic data will enable more detailed investigation of soft or incomplete selective sweeps (Malaspinas, 2016), polygenic adaptation (Buffalo & Coop, 2019, 2020), fluctuating selection (Rudman et al., 2022) and other adaptation processes.

Based on our review, temporal genomics studies have so far focused primarily on measuring the impacts of human activity on populations through direct exploitation or habitat loss. The evolutionary consequences of climate change remain comparatively understudied and these impacts represent a vital future direction for temporal genomics studies. Jensen and Leigh (2022) recently reviewed the state of the field and prospects for using temporal genomics to study the impacts of climate change on wildlife, providing several recommendations for study design that dovetail with those provided here. The parallel development of methods for using genomics to predict (mal)adaption to future climate (Bay et al., 2018) and future climate‐driven shifts in range and population size (Razgour et al., 2018) also provides a set of hypotheses that are uniquely testable using temporal genomic data and serial sampling of populations as climates change will be useful in field‐testing the accuracy of these predictive methods.

Finally, temporal genomics studies are, by definition, limited by the timing and spatial extent of the collection of suitable samples over time. Our review identified the temporal sampling scheme as key to identifying the causes of observed changes in genomic composition over time, with multiple serial samples being ideal. Extending the time horizon of ongoing temporal studies or revisiting previous studies may thus be especially useful for clarifying the causes of genetic change and the ongoing effects of environmental change on the genomic composition of populations. Appropriate preservation of samples and archiving of data will be key to assembling and integrating genomics data sets for identifying change over time. With a renewed focus on serial sampling population monitoring, temporal genomics can be both forward‐ and backward‐looking and can guide future conservation and adaptive management efforts aimed at preserving biodiversity over time.

## AUTHOR CONTRIBUTIONS

R.D.C.: formal analysis, investigation, methodology, supervision, writing—original draft; writing—review and editing; K.C.: investigation, methodology; K.S.F.: investigation, writing—review and editing; E.G.: visualization, writing—original draft, writing—review and editing; K.E.J.: investigation, writing—review and editing; B.N.R.: investigation, supervision, writing—original draft, writing—review and editing; A.S.: investigation, writing—original draft, writing—review and editing; A.A.S.: investigation, methodology, supervision, writing—review and editing; J.C.W.: methodology; M.L.P.: supervision, writing—review and editing. All authors participated in conceptualization, data curation and gave final approval for publication.

## CONFLICT OF INTEREST STATEMENT

The authors declare no conflicts of interest associated with this work.

## BENEFIT‐SHARING STATEMENT

Benefits Generated: Benefits from this research accrue from the sharing of our data and results on public databases as described above.

## Supporting information


Figures S1–S4

Appendix S1

Appendix S2

## Data Availability

Related metadata can be found on GitHub (TempGenomics‐RCN/SoF_Analyses). All data and scripts associated with the manuscript are deposited on the Dryad Digital Repository (doi: 10.5061/dryad.mkkwh714n).
