# Supplementary material for: The practice and promise of temporal genomics for measuring evolutionary responses to global change
Source: Mol Ecol Resour. 2023 Apr 2;25(5):e13789. doi: 10.1111/1755-0998.13789 (PMC12142728; doi:10.1111/1755-0998.13789)
Supplement: Supplementary file 1 — Figures S1–S4 [file MEN-25-e13789-s001.pdf]

The practice and promise of temporal genomics for measuring evolutionary responses to global change

René D. Clark, Katrina A. Catalano, Kyra S. Fitz, Eric Garcia, Kyle E. Jaynes, Brendan N. Reid, Allyson Sawkins, Anthony A. Snead, John C. Whalen, Malin L. Pinsky

SUPPLEMENTAL FIGURES

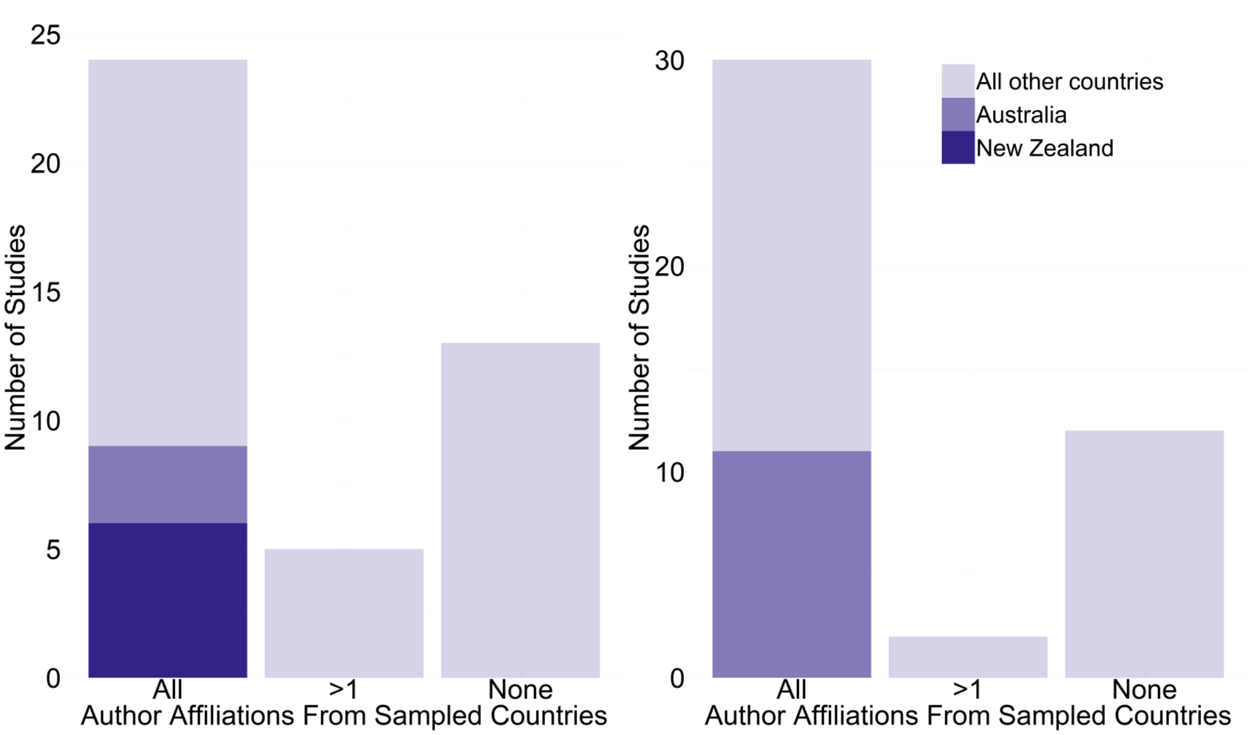

Figure S1. Number of studies that have authors primarily affiliated with all countries from which samples originated in, at least one (but not all) of the countries, or none of the countries. Studies in which samples came from Canada, W. Europe, and/or the USA are excluded from these counts. A: studies where museum samples were used, B: studies where predesigned samples were used (authors collected all samples intentionally).

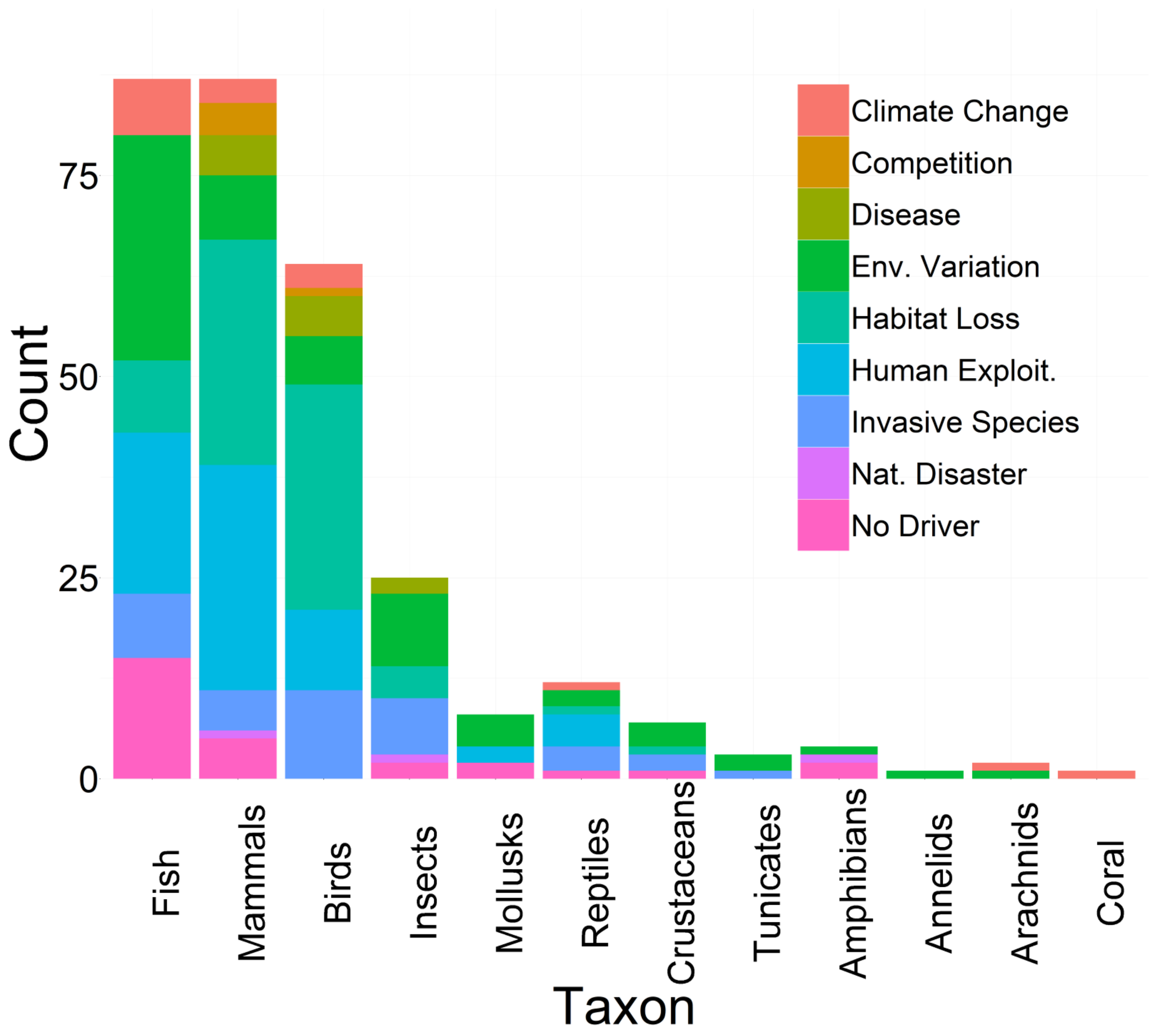

Figure S2. Distribution of temporal genomics studies across taxa by driver of change. The 16 observed taxonomic classes were condensed into higher-order taxa where appropriate. Studies could be assigned more than one driver. Studies where a driver of change could not be identified were assigned “No Driver.”

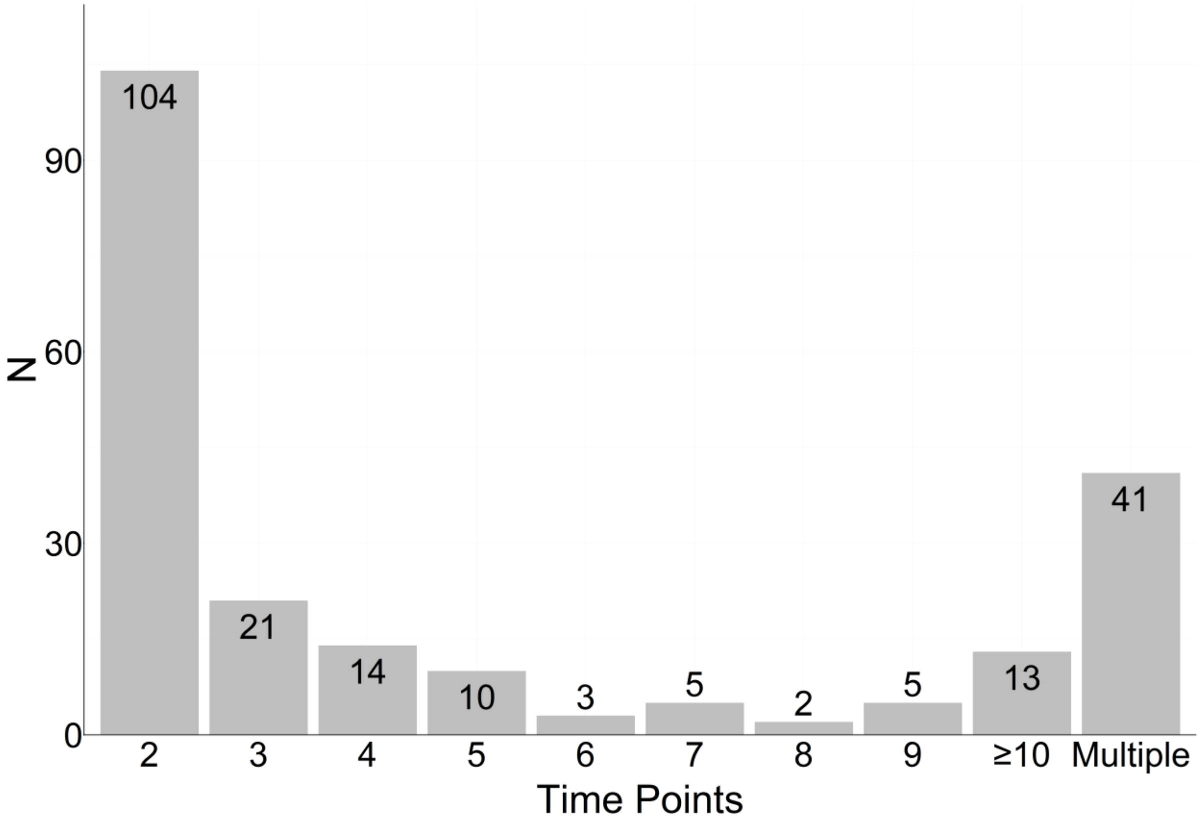

Figure S3. Distribution of temporal genomics studies across number of time points incorporated into analyses. Numbers above/below bars represent counts per time point. Studies where multiple study designs were used (e.g., some analyses or populations had 4 time points and others had 2 time points) were labeled as "Multiple."

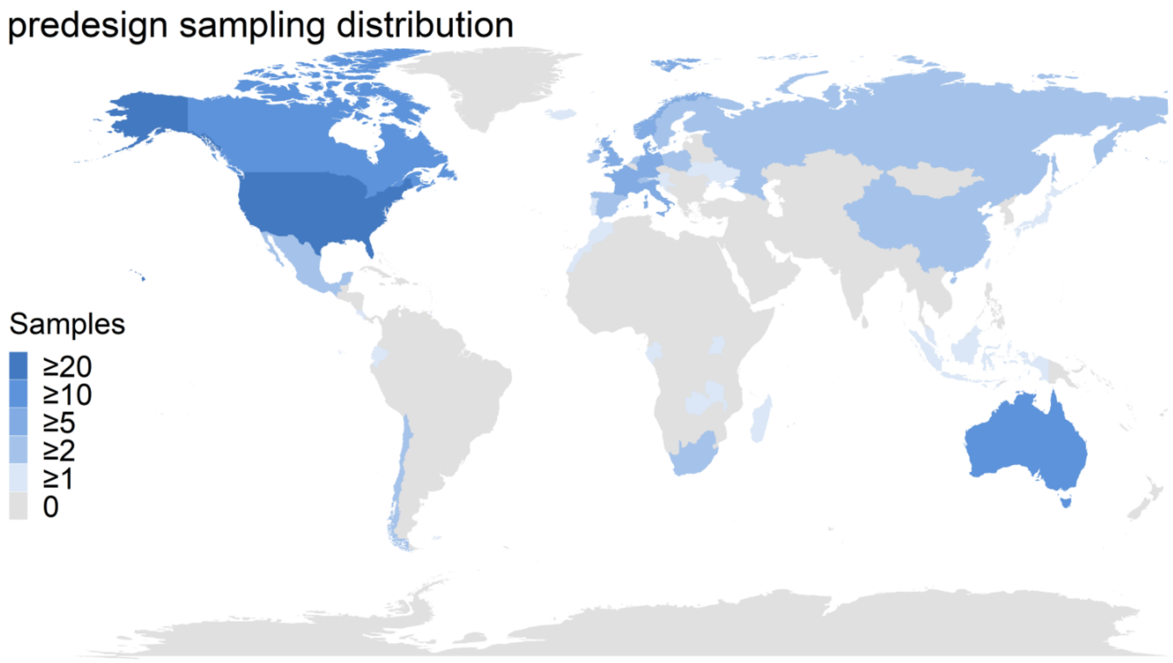

Figure S4. Distribution of countries sampled for temporal genomics studies. Studies that use museum samples are not included in the sample counts, only those where the authors collected all samples intentionally.
