# Supplementary material for: The practice and promise of temporal genomics for measuring evolutionary responses to global change
Source: Mol Ecol Resour. 2023 Apr 2;25(5):e13789. doi: 10.1111/1755-0998.13789 (PMC12142728; doi:10.1111/1755-0998.13789)
Supplement: Supplementary file 2 — Appendix S1 [file MEN-25-e13789-s003.pdf]

The practice and promise of temporal genomics for measuring evolutionary responses to 1 **global change**

René D. Clark, Katrina A. Catalano, Kyra S. Fitz, Eric Garcia, Kyle E. Jaynes, Brendan N. Reid, Allyson Sawkins, Anthony A. Snead, John C. Whalen, Malin L. Pinsky

Studies included in temporal genetics paper

**Taxon**	**Adaptation**	**Connectivity**	**Diversity**	**Population Size**	**References**
Actinopterygii	X (17)	X (41)	X (40)	X (23)	Almodovar *et al.* (2020); Anderson *et al.* (2020); Baillie *et al.* (2016); Bangs *et al.* (2018); Bekkevold *et al.* (2004); Bekkevold *et al.* (2015); Blondel *et al.* (2019); Bonanomi *et al.* (2016); Burford *et al.* (2011); Campbell *et al.* (2017); Carrier *et al.* (2020); Chassaing *et al.* (2016); Chebib *et al.* (2016); Christensen *et al.* (2018); Culumber *et al.* (2012); Dahle *et al.* (2018); Deagle *et al.* (2013); Demandt (2010); Diedericks *et al.* (2018); Faulks *et al.* (2017); Franchini *et al.* (2012); Fraser *et al.* (2007a); Fraser *et al.* (2007b); Gilbert-Horvath *et al.* (2006); Gold *et al.* (2001); Gong *et al.* (2019); Hemmer-Hansen *et al.* (2007); Hemmer-Hansen *et al.* (2019); Husemann *et al.* (2015); Hutchinson *et al.* (2003); Johnstone *et al.* (2013); Jolly *et al.* (2011); Kelson *et al.* (2020); Kerr *et al.* (2019); Knutsen *et al.* (2011); Liu *et al.* (2011); Lucentini *et al.* (2009); Maes *et al.* (2006); Mamoozadeh *et al.* (2020); Martin (2010); Mathieu-Begne *et al.* (2019); McBride *et al.* (2015); McElroy *et al.* (2011); McKeown *et al.* (2015); Osborne *et al.* (2010); Osborne *et al.* (2017); Ozerov *et al.* (2013); Pita *et al.* (2011); Pita *et al.* (2017); Poulsen *et al.* (2006); Poulsen *et al.* (2011); Priest *et al.* (2012); Pukk *et al.* (2013); Roberts *et al.* (2011); Salzburger *et al.* (2006); Serbezov *et al.* (2012); Snyder & Stepien (2017); Therkildsen *et al.* (2013)[a]; Therkildsen *et al.* (2013)[b]; Tseng *et al.* (2012); Turner *et al.* (2015); Waits *et al.* (2008); Waldman *et al.* (2019)
Amphibia		X (1)	X (3)	X (3)	Holmes (2015); Nunziata *et al.* (2015); Potvin *et al.* (2017) Rowe & Beebee (2004)
Arachnida	X (1)	X (1)	X (2)	X (1)	Krehenwinkel & Tautz (2013); van Schaik *et al.* (2014)
Ascidiacea	X (1)		X (1)		Caputi *et al.* (2019); Pérez-Portela *et al.* (2012)

Aves	X (4)	X (13)	X (33)	X (22)	Athrey *et al.* (2011); Athrey *et al.* (2012); Akiyama *et al.* (2017); Barbanera *et al.* (2015); Bergner *et al.* (2016); Bolton *et al.* (2018); Carling & Zuckerberg (2011); D'Elia *et al.* (2016); Draheim *et al.* (2012); Drovetski *et al.* (2012); Dussex *et al.* (2018); Farrington & Petren *et al.* (2011); Farrington *et al.* (2019); Foster *et al.* (2007); Groombridge *et al.* (2009); Gilroy *et al.* (2016); Harrisson *et al.* (2016); Jackson *et al.* (2016); Johnson & Dunn (2006); Johnson *et al.* (2007); Klinga *et al.* (2020); Lawson *et al.* (2017); Lounsberry *et al.* (2014); Martínez-Cruz *et al.* (2007); Morrison *et al.* (2020); Moulton *et al.* (2017); Muñoz-Fuentes *et al.* (2005)Padró *et al.* (2020); Reding *et al.* (2010); Rutkowski *et al.* (2017); Sefc *et al.* (2007); Schroeder *et al.* (2010); Sonsthagen *et al.* (2020); Sutton *et al.* (2015); Taylor *et al.* (2007); Vallianatos *et al.* (2002); Vilaça *et al.* (2012); Walsh *et al.* (2017); Wells *et al.* (2019)
Bivalvia	X (1)	X (1)	X (1)	X (1)	Sun & Hedgecock (2017)
Branchipoda		X (1)	X (1)		Griebel *et al.* (2016)
Chondrichthyes		X (1)			Thorburn *et al.* (2018)
Gastropoda	X (2)	X (4)	X (5)	X (4)	Charbonnel *et al.* (2002); Coates *et al.* (2014); Hawk & Geller *et al.* (2019); Lamy *et al.* (2012); Lee & Boulding (2009); Rhode *et al.* (2017)
Hexacorallia		X (1)	X (1)		Underwood *et al.* (2018)
Hexanauplia		X (1)			Van Wormhoudt (2015)
Insecta	X (1)	X (12)	X (14)	X (6)	Anderson *et al.* (2008); Baudouin *et al.* (2017); Chen *et al.* (2010); Cogni *et al.* (2014); Fountain *et al.* (2018); Gauthier *et al.* (2020); Gradish *et al.* (2015); Gredler *et al.* (2015); Lalonde & Marcus (2020); Larroque *et al.* (2019); Lemic *et al.* (2013); Leo *et al.* (2017); Perera *et al.* (2020); Rangel *et al.* (2020); Schmid *et al.* (2018); Solorzano *et al.* (2010); Stauffer-Olsen *et al.* (2017); Suni *et al.* (2014); Thompson *et al.* (2020); van Schaik *et al.* (2015); Wolf *et al.* (2012)
Malacostraca	X (1)	X (3)	X (4)	X (1)	Cabezas *et al.* (2019); da Silva & Tolley (2018); Lehnert *et al.* (2018); Papetti *et al.* (2005); Pavesi *et al.* (2011)
Mammalia	X (4)	X (30)	X (46)	X (29)	Arora *et al.* (2012); Bi *et al.* (2013); Bonnet & Postma (2018); Cammen *et al.* (2018)[a]; Cammen *et al.* (2018)[b]; Carroll *et al.* (2019); Cullingham & Moehrenschlager (2013); De Barba *et al.* (2010); Devillard *et al.* (2011); Draheim *et al.* (2018); Fietz *et al.* (2016);

					Floyd *et al.* (2011); Gagne *et al.* (2018); García-Navas *et al.* (2015); Gaubert *et al.* (2019); Gervasi *et al.* (2017); Haanes *et al.* (2013); Hagemann *et al.* (2019); Hagen *et al.* (2015); Heppenheimer *et al.* (2020); Hsu *et al.* (2017); Jansson *et al.* (2012); Jordan *et al.* (2012); Kruckenhauser *et al.* (2009); Lancaster *et al.* (2006); Leonard *et al.* (2005); Leonard & Wayne (2008); Lilley *et al.* (2020); Lonsinger *et al.* (2018); Matocq & Villablanca (2001); Mikle *et al.* (2016); Mondol *et al.* (2013); Morris *et al.* (2013);Nichols *et al.* (2012); Perrine *et al.* (2007); Pertoldi *et al.* (2008)[a]; Pertoldi *et al.* (2008)[b]; Petersen *et al.* (2010); Pfau *et al.* (2019); Pichler & Baker (2000); Piggott *et al.* (2018); Pilot *et al.* (2010); Ploshnitsa *et al.* (2012); Quinn *et al.* (2019); Robins *et al.* (2016); Rodríguez *et al.* (2011); Sacks *et al.* (2010); Sarno *et al.* (2015); Skrbinšek *et al.* (2012); Stronen *et al.* (2019); Sugimoto *et al.* (2014); Tokarska *et al.* (2009); Valtonen *et al*. (2012); Vega *et al.* (2017); Volkmann *et al.* (2015); White *et al.* (2018); Xie & Zhang (2006)
Polychaeta			X (1)		Kesaniemi *et al.* (2014)
Reptilia	X (1)	X (6)	X (6)	X (3)	Edwards *et al.* (2013)Jensen *et al.* (2016); Jensen *et al.* (2018)[a]; Jensen *et al.* (2018)[b]; Katz *et al.* (2014); Leaché *et al.* (2017); Lukoschek (2018); Moule *et al.* (2015); Nishizawa *et al.* (2016)

SUPPLEMENTAL RESOURCES

Akiyama T, Momose K, Onuma M, Matsumoto F & Masuda R (2017) Low genetic variation of red-crowned cranes on Hokkaido Island, Japan, over the hundred years. *Zoological Science*, **34**, 211-216. doi:10.2108/zs160194

Almodóvar A, Leal S, Nicola GG, Hórreo JL, García-Vázquez E & Elvira B (2020) Long-term stocking practices threaten the original genetic diversity of the southernmost European populations of Atlantic salmon *Salmo salar*. *Endangered Species Research*, **41,** 303-317. doi:10.3354/ers01029

Anderson SJ, Conrad KF, Gillman MP, Woiwod IP & Freeland JR (2008) Phenotypic changes and reduced genetic diversity have accompanied the rapid decline of the garden tiger moth (*Arctia caja*) in the U.K. *Ecological Entomology*, **33**, 638-645. doi:10.1111/j.1365-2311.2008.01013.x

Anderson G, Lal M, Stockwell B ,Hampton J, Smith N, Nicol S & Rico C (2020) No population genetic structure of skipjack tuna (*Katsuwonus pelamis*) in the tropical Western and Central Pacific assessed using single nucleotide polymorphisms. *Frontiers in Marine Science*, 1102.

doi:10.3389/fmars.2020.570760

Arora N, Van Noordwijk MA, Ackermann C, Willems EP, Nater A, Greminger M, Nietlisbach P, Dunkel LP, Atmoko SSU, Pamungkas J, Perwitasari-Farajallah D, Van Schaik CP & Krützen M (2012) Parentage-based pedigree reconstruction reveals female matrilineal clusters and male-biased dispersal in nongregarious Asian great apes, the Bornean orang-utans (*Pongo pygmaeus*). *Molecular Ecology*, **21**, 3352-3362. doi:10.1111/j.1365-294X.2012.05608.x

Athrey G, Barr KR, Lance RF & Leberg PL (2012) Birds in space and time: Genetic changes accompanying anthropogenic habitat fragmentation in the endangered black-capped vireo (*Vireo atricapilla*). *Evolutionary Applications*, **5**, 540-552. doi:10.1111/j.1752-4571.2011.00233.x

Athrey G, Lindsay DL, Lance RF & Leberg PL (2011) Crumbling diversity: Comparison of historical archived and contemporary natural populations indicate reduced genetic diversity and increasing genetic differentiation in the golden-cheeked warbler. *Conservation Genetics*, **12**, 1345-1355. doi:10.1007/s10592-011-0235-8

Baillie SM, Muir AM, Scribner K, Bentzen P & Krueger CC (2016) Loss of genetic diversity and reduction of genetic distance among lake trout *Salvelinus namaycush* ectomorphs, Lake Superior 1959 to 2013. *Journal of Great Lakes Research*, **42**, 204-216. doi:10.1016/j.glr.2016.02.001

Bangs MR, Oswald KJ, Greig TW, Leitner JK, Rankin DM & Quattro JM (2018) Introgressive hybridization and species turnover in reservoirs: A case study involving endemic and invasive basses (Centrarchidae: *Micropterus*) in southeastern North America. *Conservation Genetics*, **19**, 57-69. doi:10.1007/s10592-017-1018-7

Barbanera F, Forcina G, Cappello A, Guerrini M, van Grouw H & Aebischer NJ (2015) Introductions over introductions: The genomic adulteration of an early genetically valuable alien species in the United Kingdom. *Biological Invasions*, **17**, 409-422. doi:10.1007/s10530-014-0739-5

Bass AL, Epperly SP & Braun-McNeill J (2004) Multi-year analysis of stock composition of a loggerhead turtle (*Caretta caretta*) foraging habitat using maximum likelihood and Bayesian methods. *Conservation Genetics*, **5**, 783-796. doi:10.1007/s10592-004-1979-1

Baudouin G, Dedeine F, Bech N, Bankhead-Dronnet S, Dupont S & Bagnères A (2017) An American termite in Paris: Temporal colony dynamics. *Genetica*, **145**, 491-502. doi:10.1007/s10709-017-9991-9

Bekkevold D, Hansen MM & Mensberg KD (2004) Genetic detection of sex-specific dispersal in historical and contemporary populations of anadramous brown trout *Salmo trutta*. *Molecular Ecology*, **13**, 1707-1712. doi:10.1111/j.1365-294X.2004.02156.x

Bekkevold D, Jacobsen L, Hemmer-Hansen J, Berg S & Skov C (2015) From regionally predictable to locally complex population structure in a

freshwater top predator: River systems are not always the unit of connectivity in Northern Pike *Esox lucius*. *Ecology of Freshwater Fish*, **24**, 305-316. doi:10.1111/eff.12149

Bergner LM, Dussex N, Jamieson IG & Robertson BC (2016) European colonization, not Polynesian arrival, impacted population size and genetic diversity in the critically endangered New Zealand Kākāpō. *Journal of Heredity*, **107**, 593-602. doi:10.1093/jhered/esw065

Bi K, Linderoth T, Vanderpool D, Good JM, Nielsen R & Moritz C (2013) Unlocking the vault: Next-generation museum population genomics. *Molecular Ecology*, **22**, 6018-6032. doi:10.1111/mec.12516

Blondel L, Baillie L, Quinton J, Alemu JB, Paterson I, Hendry AP & Bentzen P (2019) Evidence for contemporary and historical gene flow between guppy populations in different watersheds, with a test for associations with adaptive traits. *Ecology and Evolution*, **9**, 4504-4517. doi:10.1002/ece3.5033

Bolton PE, Rollins LA, Brazill-Boast J, Maute KL, Legge S, Austin JJ & Griffith SC (2018) Genetic diversity through time and space: Diversity and demographic history from natural history specimens and serially sampled contemporary populations of the threatened Gouldian finch (*Erythrura gouldiae*). *Conservation Genetics*, **19**, 737-754. doi:10.1007/s10592-018-1051-1

Bonanomi S, Therkildsen NO, Retzel A, Hedeholm RB, Pedersen MW, Meldrup D, Pampoulie C, Hemmer-Hansen J, Grønkjær P & Nielsen EE (2016) Historical DNA documents long-distance natal homing in marine fish. *Molecular Ecology*, **25**, 2727-2734. doi:10.1111/mec.13580

Bonnet T & Postma E (2018) Fluctuating selection and its (elusive) evolutionary consequences in a wild rodent population. *Journal of Evolutionary Biology*, **31**, 572-586. doi:10.5061/dryad.6767m

Burford MO, Carr MH & Bernardi G (2011) Age-structured genetic analysis reveals temporal and geographic variation within and between two cryptic rockfish species. *Marine Ecology Progress Series*, **442**, 201-215. doi:10.3354/meps09329

Cabezas MP, Ros Clemente M, dos Santos AM, Martinez-Laiz G, Xavier R, Montelli L, Hoffman R, Fersi A, Dauvin JC & Guerra-Garcia JM (2019) Unravelling the origin and introduction pattern of the tropical species *Paracaprella pusilla* Mayer 1890 (*Crustacea, Amphipoda, Caprellidae*) in temperate European waters: First molecular insights from a spatial and temporal perspective. *NeoBiota*, **47**, 43-80. doi:10.3897/neobiota.47.32408

Cammen KM, Schultz TF, Bowen WD, Hammill MO, Puryear WB, Runstadler J, Wenzel FW, Wood SA & Kinnison M (2018a) Genomic signatures of population bottleneck and recovery in Northwest Atlantic pinnipeds. *Ecology and Evolution*, **8**, 6599-6614. doi:10.1002/ece3.4143

Cammen KM, Vincze S, Heller AS, Mcleod BA, Wood SA, Bowen WD, Hammill MO, Puryear WB, Runstadler J, Wenzel FW, Kinnison MK

& Frasier TR (2018b) Genetic diversity from pre-bottleneck to recovery in two sympatric pinniped species in the Northwest Atlantic. *Conservation Genetics*, **19**, 555-569. doi:10.1007/s10592-017-1032-9

Campbell NR, Kamphaus C, Murdoch K & Narum SR (2017) Patterns of genomic variation in Coho salmon following reintroduction to the interior Columbia River. *Ecology and Evolution*, **7**, 10350-10360. doi:10.1002/ece3.3492

Caputi L, Toscano F, Arienzo M, Ferrara L, Procaccini G & Sordino P (2019) Temporal correlation of population composition and environmental variables in the marine invader *Ciona robusta*. *Marine Ecology*, **40**, e12543. doi:10.1111/maec.12543

Carling MD & Zuckerberg B (2011) Spatio-temporal changes in the genetic structure of the *Passerina* bunting hybrid zone. *Molecular Ecology*, **20**, 1166-1175. doi:10.1111/j.1365-294X.2010.04987.x

Carrier E, Ferchaud A, Normandeau E, Sirois P & Bernatchez L (2020) Estimating the contribution of Greenland halibut (*Reinhardtius hippoglossoides*) stocks to nurseries by means of genotyping-by-sequencing: Sex and time matter. *Evolutionary Applications*, **13**, 2155-2167. doi:10.1111/eva.12979

Carroll RP, Litvaitis MK, Clements SJ, Stevens CL & Litvaitis JA (2019) History matters: Contemporary versus historic population structure of bobcats in the New England region, USA. *Conservation Genetics*, **20**, 734-757. doi:10.1007/s10592-019-01170-8

Charbonnel N, Quesnoit M, Razatavonjizay R, Brémond P, Jarne P & Ims RA (2002) A spatial and temporal approach to microevolutionary forces affecting population biology in the freshwater snail *Biomphalaria pfeifferi*. *The American Naturalist*, **160**, 741-755. doi:10.1086/343875

Chassaing O, Desse-Berset N, Hänni C, Hughes S & Berrebi P (2016) Phylogeography of the European sturgeon (*Acipenser sturio*): A critically endangered species. *Molecular Phylogenetics and Evolution*, **94**, 346-357. doi:10.1016/j.ympev.2015.09.020

Chebib J, Renaut S, Bernatchez L & Rogers SM (2016) Genetic structure and within-generation genome scan analysis of fisheries-induced evolution in a lake whitefish (*Coregonus clupeaformis*) population. *Conservation Genetics*, **17**, 473-483. doi:10.1007/s10592-015-0797-y

Chen YH, Berlocher SH, Opp SB & Roderick GK (2010) Post-colonization temporal genetic variation of an introduced fly, *Rhagoletis completa*. *Genetica*, **138**, 1059-1075. doi:10.1007/s10709-010-9491-7

Christensen C, Jacobsen MW, Nygaard R & Hansen MM (2018) Spatiotemporal genetic structure of anadromous Arctic char (*Salvelinus alpinus*) populations in a region experiencing pronounced climate change. *Conservation Genetics*, **19**, 687-700. doi:10.1007/s10592-018-1047-x

Coates JH, Hovel KA, Butler JL & Bohonak AJ (2014) Recruitment and recovery of pink abalone (*Haliotis corrugate*) in a historically overexploited kelp forest: Are local populations self-sustaining? *Journal of Experimental Marine Biology and Ecology*, **460**, 184-192.

doi:10.1016/j.jembe.2014.07.004

Cogni R, Kuczynski C, Koury S, Lavington E, Behrman EL, O'Brien KR, Schmidt PS & Eanes WF (2014) The intensity of selection acting on the *couch potato* gene – spatial-temporal variation in a diapause cline. *Evolution*, **68**, 538-548. doi:10.1111/evo.12291

Cullingham CI & Moehrenschlager A (2013) Temporal analysis of genetic structure to assess population dynamics of reintroduced swift foxes. *Conservation Biology*, **27**, 1389-1398. doi:10.1111/cobi.12122

Culumber ZW, Shepard DB, Coleman SW, Rosenthal GG & Tobler M (2012) Physiological adaptation along environmental gradients and replicated hybrid zone structure in swordtails (Teleostei: *Xiphorphorus*). *Journal of Evolutionary Biology*, **25**, 1800-1814. doi:10.1111/j.1420-9101.2012.02562.x

D'Elia J, Haig SM, Mullins TD & Miller MP (2016) Ancient DNA reveals substantial genetic diversity in the California condor (*Gymnogyps californianus*) prior to a population bottleneck. *The Condor*, **118**, 703-714. doi:10.1650/CONDOR-16-35.1

da Silva JM & Tolley KA (2018) Conservation genetics of an endemic and threatened amphibian (*Capensibufo rosei*): A leap towards establishing a genetic monitoring framework. *Conservation Genetics*, **19**, 349-363. doi:10.1007/s10592-017-1008-9

Dahle G, Johansen T, Westgaard J, Aglen A & Glover KA (2018) Genetic management of mixed-stock fisheries "real-time": The case of the largest remaining cod fishery operating in the Atlantic in 2007-2017. *Fisheries Research*, **205**, 77-85. doi:10.1016/j.fishres.2018.04.006

De Barba M, Waits LP, Garton EO, Genovesi P, Randi E, Mustoni A & Groff C (2010) The power of genetic monitoring for studying demography, ecology and genetics of a reintroduced brown bear population. *Molecular Ecology*, **19**, 3938-3951. doi:10.1111/j.1365-294X.2010.04791.x

Deagle BE, Jones FC, Abesher DM, Kingsley DM & Reimchen TE (2013) Phylogeography and adaptation genetics of stickleback from the Haida Gwaii archipelago revealed using genome-wide single nucleotide polymorphism genotyping. *Molecular Ecology*, **22**, 1917-1932. doi:10.1111/mec.12215

Demandt MH (2010) Temporal changes in genetic diversity of isolated populations of perch and roach. *Conservation Genetics*, **11**, 249-255. doi:10.1007/s10592-009-0027-6

Devillard S, Santin-Janin H, Say L & Pontier D (2011) Linking genetic diversity and temporal fluctuations in population abundance of the introduced feral cat (*Felis silvestris catus*) on the Kerguelen archipelago. *Molecular Ecology*, **20**, 5141-5153. doi:10.1111/j.1365-294X.2011.05329.x

Diedericks G, Henriques R, von der Heyden S, Weyl OLF & Hui C (2018) The ghost of introduction past: Spatial and temporal variability in the genetic diversity of invasive smallmouth bass. *Evolutionary Applications*, **11**, 1609-1629. doi:10.1111/eva.12652

Draheim HM, Baird P & Haig SM (2012) Temporal analysis of mtDNA variation reveals decreased genetic diversity in least terns. *The Condor*, **114**, 145-154. doi:10.1525/cond.2012.110007

Draheim HM, Moore JA, Fortin M & Scribner KT (2018) Beyond the snapshot: Landscape genetic analysis of time series data reveal responses of American black bears to landscape change. *Evolutionary Applications*, **11**, 1219-1230. doi:10.1111/eva.12617

Drovetski SV, Kitaysky AS, Mode NA, Zink RM, Igbal U & Barger C (2012) mtDNA haplotypes differ in their probability of being eliminated by a mass die-off in an abundant seabird. *Heredity*, **109**, 29-33.

Dussex N, Von Seth J, Robertson BC & Dalén L (2018) Full mitogenomes in the critically endangered Kākāpō reveal major post-glacial and anthropogenic effects on neutral genetic diversity. *Genes*, **9**, 220. doi:10.3390/genes9040220

Farrington HL & Petren K (2011) A century of genetic change and metapopulation dynamics in the Galápagos warbler finches (*Certhidea*). *Evolution*, **65**, 3148-3161. doi:10.1111/j.1558-5646.2011.01385.x

Farrington HL, Lawson LP & Petren K (2019) Predicting population extinctions in Darwin's finches. *Conservation Genetics*, **20**, 825-836. doi:10.1007/S10592-019-01175-3

Faulks LK, Kerezsy A, Unmack PJ, Johnson JB & Hughes JM (2017) Going, going, gone? Loss of genetic diversity in two critically endangered Australian freshwater fishes, *Scaturiginichthys vermeilipinnis* and *Chlamydogobius squamigenus*, from Great Artesian Basin springs at Edgbaston, Queensland, Australia. *Aquatic Conservation - Marine and Freshwater Ecosystems*, **27**, 39-50. doi:10.1002/aqc.2684

Fietz K, Galatius A, Teilmann J, Dietz R, Frie AK, Klimova A, Palsbøll PJ, Jensen LF, Graves JA, Hoffman JI, Olsen MT (2016) Shift of grey seal subspecies boundaries in response to climate, culling and conservation. *Molecular Ecology*, **25**, 4097-4112. doi:10.111/mec.13748

Floyd CH, Van Vuren DH, Crooks KR, Jones KL, Garcelon DK, Belfiore NM, Dragoo JW & May B (2011) Genetic differentiation of island spotted skunks, *Spilogale gracilis amphiala*. *Journal of Mammalogy*, **92**, 148-158. doi:10.1644/09-MAMM-A-204.1

Foster JT, Woodworth BL, Eggert LE, Hart PJ, Palmer D, Duffy DC & Fleischer RC (2007) Genetic structure and evolved malaria resistance in Hawaiian honeycreepers. *Molecular Ecology*, **16**, 4738-4746. doi:10.1111/j.1365-294X.2007.03550.x

Fountain T, Husby A, Nonaka E, DiLeo MF, Korhonen HJ, Rastas P, Schulz T, Saastamoinen M & Hanski I (2018) Inferring dispersal across a fragmented landscape using reconstructed families in the Glanville fritillary butterfly. *Evolutionary Applications*, **11**, 287-297.

doi:10.1111/eva.12552

Franchini P, Sola L, Crosetti D, Milana V & Rossi AR (2012) Low levels of population genetic structure in the gilthead sea bream, *Sparus aurata*, along the coast of Italy. *ICES Journal of Marine Science*, **69**, 41-50. doi:10.1093/icesjms/fsr175

Fraser DJ, Hansen MM, Østergaard S, Tessier N, Legault M & Bernatchez L (2007a) Comparative estimation of effective population sizes and temporal gene flow in two contrasting population systems. *Molecular Ecology*, **16**, 3866-3889. doi:10.1111/j.1365-294X.2007.03453.x

Fraser DJ, Jones MW, McParland TL & Hutchings JA (2007b) Loss of historical immigration and the unsuccessful rehabilitation of extirpated salmon populations. *Conservation Genetics*, **8**, 527-546. doi:10.1007/s10592-006-9188-8

Gagne RB, Tinker MT, Gustafson KD, Ralls K, Larson S, Tarjan LM, Miller MA & Ernest HB (2018) Measures of effective population size in sea otters reveal special considerations for wide-ranging species. *Evolutionary Applications*, **11**, 1779-1790. doi:10.1111/eva.12642

García-Navas V, Bonnet T, Waldvogel D, Wandeler P, Camenisch G & Postma E (2015) Gene flow counteracts the effect of drift in a Swiss population of snow voles fluctuating in size. *Biological Conservation*, **191**, 1680177. doi:10.1016/j.biocon.2015.06.021

Gaubert P, Justy F, Mo G, Aguilar A, Danyer E, Borrell A, Dendrinos P, Öztürk B, Improta R, Tonay AM & Karamanlidis AA (2019) Insights from 180 years of mitochondrial variability in the endangered Mediterranean monk seal (*Monachus monachus*). *Marine Mammal Science*, **35**, 1489-1511. doi:10.1111/mms.12604

Gauthier J, Pajkovic M, Neuenschwander S, Kaila L, Schmid S, Orlando L & Alvarez N (2020) Museomics identifies genetic erosion in two butterfly species across the 20th century in Finland. *Molecular Ecology Resources*, **20**, 1191-1205. doi:10.1111/1755-0998.13167

Gervasi V, Boitani L, Paetkau D, Posillico M, Randi E & Ciucci P (2017) Estimating survival in the Apennine brown bear accounting for uncertainty in age classification. *Population Ecology*, **59**, 119-130. doi:10.1007/s10144-017-0587-0

Gilbert-Horvath EA, Larson RJ & Garza JC (2006) Temporal recruitment patterns and gene flow in kelp rockfish (*Sebastes atrovirens*). *Molecular Ecology*, **15**, 3801-3815. doi:10.1111/j.1365-294X.2006.03033.x

Gilroy D, van Oosterhout C, Komdeur J & Richardson DS (2016) Avian β-defensin variation in bottlenecked populations: The Seychelles warbler and other congeners. *Conservation Genetics*, **17**, 661-674. doi:10.1007/s10592-016-0813-x

Gold JR, Burridge CP & Turner TF (2001) A modified stepping-stone model of population structure in red drum, *Sciaenops ocellatus* (Sciaenidae), from the northern Gulf of Mexico. *Genetica*, **111**, 305-317. doi:10.1023/A:1013705230346

Gong X, Davenport ER, Wang D & Clark AG (2019) Lack of spatial and temporal genetic structure of Japanese eel (*Anguilla japonica*) populations. *Conservation Genetics*, **20**, 467-475. doi:10.1007/s10592-019-01146-8

Gradish AE, Keyghobadi N & Otis GW (2015) Population genetic structure and genetic diversity of the threatened White Mountain arctic butterfly (*Oeneis melissa semidea*). *Conservation Genetics*, **16**, 1253-1264. doi:10.1007/s10592-015-0736-y

Gredler JN, Hish AJ & Noor MAF (2015) Temporal stability of molecular diversity measures in natural populations of *Drosophila pseudoobscura* and *Drosophila persimilis*. *Journal of Heredity*, **106**, 407-411. doi:10.1093/jhered/esv027

Griebel J, Gieβler S, Yin M & Wolinska J (2016) Parental and hybrid *Daphnia* from the *D. longispina* complex: Long-term dynamics in genetic structure and significance of overwintering modes. *Journal of Evolutionary Biology*, **29**, 810-823. doi:10.1111/jeb.12828

Groombridge JJ, Dawson DA, Burke T, Prys-Jones R, Brooke ML & Shah N (2009) Evaluating the demographic history of the Seychelles kestrel (*Falco araea*): Genetic evidence for recovery from a population bottleneck following minimal conservation management. *Biological Conservation*, **142**, 2250-2257. doi:10.1016/j.biocon.2009.04.026

Haanes H, Rosvold J & Røed KH (2012) Non-indigenous introgression into the Norwegian red deer population. *Conservation Genetics*, **14**, 237-242. doi:10.1007/s10592-012-0431-1

Hagemann L, Arandjelovic M, Robbins MM, Deschner T, Lewis M, Froese G, Boesch C & Vigilant L (2019) Long-term inference of population size and habitat use in a socially dynamic population of wild western lowland gorillas. *Conservation Genetics*, **20**, 1303-1314. doi:10.1007/s10592-019-01209-w

Hagen SB, Kopatz A, Aspi J, Kojola I & Eiken HG (2015) Evidence of rapid change in genetic structure and diversity during range expansion in a recovering large terrestrial carnivore. *Proceedings of the Royal Society B: Biological Sciences*, **282**, 20150092. doi:10.1098/rspb.2015.0092

Harrisson KA, Pavlova A, Gonçalves da Silva A, Rose R, Bull JK, Lancaster ML, Murray N, Quin B, Menkhorst P, Magrath MJL, Sunnucks P (2016) Scope for genetic rescue of an endangered subspecies though re-establishing natural gene flow with another subspecies. *Molecular Ecology*, **25**, 1242-1258. doi:10.1111/mec.13547

Hawk HL & Geller JB (2018) DNA entombed in archival seashells reveal low historical mitochondrial genetic diversity of endangered white abalone *Haliotis sorenseni*. *Marine and Freshwater Research*, **70**, 359-370. doi:10.1071/MF17342

Hemmer-Hansen J, Hüssy K, Baktoft H, Huwer B, Bekkevold D, Haslob H, Herrman J, Hinrichsen H, Krumme U, Mosegaard H, Nielsen EE, Reusch TBH, Storr-Paulsen M, Velasco A, von Dewitz B, Dierking J & Eero M (2019) Genetic analyses reveal complex dynamics within

a marine fish management area. *Evolutionary Applications*, **12**, 830-844. doi:10.1111/eva.12760

Hemmer-Hansen J, Nielsen EE, Grønkær P & Loeschcke V (2007) Evolutionary mechanisms shaping the genetic population structure of marine fishes; lessons from the European flounder (*Platichthys flesus* L.). *Molecular Ecology*, **16**, 3104-3118. doi:10.1111/j.1365-294X.2007.003367.x

Heppenheimer E, Brzeski KE, Hinton JW, Chamberlain MJ, Robinson J, Wayne RK vonHoldt BM (2020) A genome-wide perspective on the persistence of red wolf ancestry in southeastern canids. *Journal of Heredity*, **111**, 277-286. doi:10.1093/jhered/esaa006

Hess JE & Matala AP (2014) Archival genetic analysis suggests recent immigration has altered a population of Chinook salmon in an unsupplemented wilderness area. *Conservation Genetics*, **15**, 387-403. doi:10.1007/s10592-013-0546-z

Holmes I (2015) Temporal population genetic instability in range-edge western toads, *Anaxyrus boreas*. *Journal of Heredity*, **106**, 45-56. doi:10.1093/jhered/esu068

Hsu JL, Kam S, Tammone MN, Lacey EA & Hadly EA (2017) Rapid increase in genetic diversity in an endemic Patagonian tuco-tuco following a recent volcanic eruption. *Journal of Mammalogy*, **98**, 779-792. doi:10.1093/jmammal/gyx008

Husemann M, Nguyen R, Ding B & Danley PD (2015) A genetic demographic analysis of Lake Malawi rock-dwelling cichlids using spatio-temporal sampling. *Molecular Ecology*, **24**, 2686-2701. doi:10.1111/mec.13205

Hutchinson WF, van Oosterhout C, Rogers SI & Carvalho GR (2003) Temporal analysis of archived samples indicates marked genetic changes in declining North Sea cod (*Gadus morhua*). *Proceedings of the Royal Society B: Biological Sciences*, **270**, 2125-2132. doi:10.1098/rspb.2003.2493

Jackson HA, Bunbury N, Przelomska N & Groombridge JJ (2016) Evolutionary distinctiveness and historical decline in genetic diversity in the Seychelles Black Parrot *Coracopsis nigra barklyi*. *IBIS: International Journal of Avian Science*, **158**, 380-394. doi:10.1111/ibi.12343

Jansson E, Ruokonen M, Kojola I & Aspi J (2012) Rise and fall of a wolf population: Genetic diversity and structure during recovery, rapid expansion and drastic decline. *Molecular Ecology*, **21**, 5178-5193. doi:10.1111/mec.12010

Jensen MP, Bell I, Limpus CJ, Hamann M, Ambar S, Whap T, David C & FitzSimmons NN (2016) Spatial and temporal genetic variation among size classes of green turtles (*Chelonia mydas*) provides information on oceanic dispersal and population dynamics. *Marine Ecology Progress Series*, **543**, 241-256. doi:10.3354/meps11521

Jensen EL, Edwards DL, Garrick RC, Miller JM, Gibbs JP, Cayot LJ, Tapia W, Caccone A & Russello MA (2018a) Population genomics

through time provides insights into the consequences of decline and rapid demographic recovery through head-starting in a Galapagos giant tortoise. *Evolutionary Applications*, **11**, 1811-1821. doi:10.1111/eva.12682

Jensen EL, Miller JM, Edwards DL, Garrick RC, Tapia W, Caccone A & Russello MA (2018b) Temporal mitogenomics of the Galapagos Giant Tortoise from Pinzón reveals potential biases in population genetic inference. *Journal of Heredity*, **109**, 631-640. doi:10.1093/jhered/esy016

Johnson JA & Dunn PO (2006) Low genetic variation in the heath hen prior to extinction and implications for the conservation of prairie-chicken populations. *Conservation Genetics*, **7**, 37-48. doi:10.1007/s10592-005-7856-8

Johnson JA, Dunn PO & Bouzat JL (2007) Effects of recent population bottlenecks on reconstructing the demographic history of prairie-chickens. *Molecular Ecology*, **16**, 2203-2222. doi:10.1111/j.1365-294X.2007.03285.x

Johnstone DL, O'Connell MF, Palstra FP & Ruzzante DE (2013) Mature male parr contribution to the effective size of an anadromous Atlantic salmon (*Salmo salar*) population over 30 years. *Molecular Ecology*, **22**, 2394-2407. doi:10.1111/mec.12186

Jolly MT, Paitland PS & Genner MJ (2011) Genetic monitoring of two decades of hybridization between allis shad (*Alosa alosa*) and twaite shad (*Alosa fallax*). *Conservation Genetics*, **12**, 1087-1100. doi:10.1007/s10592-011-0211-3

Jordan NR, Messenger J, Turner P, Croose E, Birks J & O'Reilly C (2012) Molecular comparison of historical and contemporary pine marten (*Martes martes*) populations in the British Isles: Evidence of differing origins and fates, and implications for conservation management. *Conservation Genetics*, **13**, 1195-1212. doi:10.1007/s10592-012-0365-7

Katz EM, Bishop JM & Tolley KA (2014) Temporal changes in allelic variation among Cape Dwarf Chameleons, *Bradypodion pumilum*, inhabiting a transformed, semi-urban wetland. *African Journal of Herpetology*, **63**. doi:10.1080/21564574.2013.834852

Kelson SJ, Miller MR, Thompson TQ, O'Rourke SM & Carlson SM (2020) Temporal dynamics of migration-linked genetic variation are driven by streamflows and riverscape permeability. *Molecular Ecology*, **29**, 870-885. doi:10.1111/mec.15367

Kerr Q, Fuentes-Pardo AP, Kho J, McDermid JL & Ruzzante DE (2019) Temporal stability and assignment power of adaptively divergent genomic regions between herring (*Clupea harengus*) seasonal spawning aggregations. *Ecology and Evolution*, **9**, 500-510. doi:10.1002/ece3.4768

Kesäniemi J, Mustonen M, Boström C, Hansen BW & Knott KE (2014) Temporal genetic structure in a poecilogonous polychaete: The interplay of developmental mode and environmental stochasticity. *BMC Evolutionary Biology*, **14**, 12. doi:10.1186/1471-2148-14-12

Klinga P, Mikoláš M, Delegan IV, Dănilă G, Urban P, Paule L & Kaňuch P (2020) Temporal landscape genetic data indicate an ongoing disruption of gene flow in a relict bird species. *Conservation Genetics*, **21**, 329-340. doi:10.1007/s10592-020-01253-x

Knutsen H, Olsen EM, Jorde PE, Espeland SH, André C & Stenseth NC (2011) Are low but statistically significant levels of genetic differentiation in marine fishes 'biologically meaningful'? A case study of coastal Atlantic cod. *Molecular Ecology*, **20**, 768-783. doi:10.1111/j.1365-294X.2010.04979.x

Krehenwinkel H & Tautz D (2013) Northern range expansion of European populations of the wasp spider *Argiope bruennichi* is associated with global warming – correlated genetic admixture and population-specific temperature adaptations. *Molecular Ecology*, **22**, 2232-2248. doi:10.1111/mec.12223

Kruckenhauser L, Rauer G, Däubl B & Haring E (2009) Genetic monitoring of a founder population of brown bears (*Ursus arctos*) in central Austria. *Conservation Genetics*, **10**, 1223-1233. doi:10.1007/s10592-008-9654-6

Lalonde MML & Marcus JM (2020) Back to the future: Updates on the invasion history of *Junonia* butterflies in Florida and the mystery of Chokoloskee. *The Journal of the Lepidopterists' Society*, **74**, 83-94. doi:10.18473/lepi.74i2.a3

Lamy T, Pointier JP, Jarne P & David P (2012) Testing metapopulation dynamics using genetic, demographic and ecological data. *Molecular Ecology*, **21**, 1394-1410. doi:10.1111/j.1365-294X.2012.05478.x

Lancaster ML, Gemmell NJ, Negro S, Goldsworthy S & Sunnucks P (2006) Ménage à trois on Macquarie Island: Hybridization among three species of fur seal (*Arctocephalus* spp.) following historical population extinction. *Molecular Ecology*, **15**, 3681-3692. doi:10.1111/j.1365-294X.2006.03041.x

Larroque J, Legault S, Johns R, Lumley L, Cusson M, Renaut S, Levesque RC & James PMA (2019) Temporal variation in spatial genetic structure during population outbreaks: Distinguishing among different potential drivers of spatial synchrony. *Evolutionary Applications*, **12**, 1931-1945. doi:10.1111/eva.12852

Lawson LP, Fessl B, Vargas FH, Farrington HL, Cunninghame HF, Mueller JC, Nemeth E, Sevilla PC & Petren K (2017) Slow motion extinction: Inbreeding, introgression, and loss in the critically endangered mangrove finch (*Camarhynchus heliobates*). *Conservation Genetics*, **18**, 159-170. doi:10.1007/s10592-016-0890-x

Leaché AD, Grummer JA, Harris RB & Breckheimer IK (2017) Evidence for concerted movement of nuclear and mitochondrial clines in a lizard hybrid zone. *Molecular Ecology*, **26**, 2306-2316. doi:10.1111/mec.14033

Lee HJ & Boulding EG (2009) Spatial and temporal population genetic structure of four northeastern Pacific littorinid gastropods: The effect of

mode of larval development on variation at one mitochondrial and two nuclear DNA markers. *Molecular Ecology*, **18**, 2165-2184. doi:10.1111/j.1365-294X.2009.04169.x

Lehnert SJ, DiBacco C, Jeffery NW, Blakeslee AMH, Isaksson J, Roman J, Wringe BF, Stanley RRE, Matheson K, McKenzie CH, Hamilton LC & Bradbury IR (2018) Temporal dynamics of genetic clines of invasive European green crab (*Carcinus maenas*) in eastern North America. *Evolutionary Applications*, **11**, 1656-1670. doi:10.1111/eva.12657

Lemic D, Mikac KM & Bažok R (2013) Historical and contemporary population genetics of the invasive western corn rootworm (Coleoptera: Chrysomelidae) in Croatia. *Environmental Entomology*, **42**, 811-819. doi:10.1603/EN12351

Leo SST, Gonzalez A & Millien V (2016) The genetic signature of range expansion in a disease vector – the black-legged tick. *Journal of Heredity*, **108**, 176-183. doi:10.1093/jhered/esw073

Leonard JA, Vilà C & Wayne RK (2005) Legacy lost: Genetic variability and population size of extirpated US gray wolves. *Molecular Ecology*, **126**, 198-206.

Leonard JA & Wayne RK (2008) Native Great Lakes wolves were not restored. *Biology Letters*, **4**. doi:10.1098/rsbi.2007.0354

Lilley TM, Wilson IW, Field KA, Reeder DM, Vodzak ME, Turner GG, Kurta A, Blomberg AS, Hoff S, Herzog CJ, Sewall BJ & Paterson S (2020) Genome-wide changes in genetic diversity in a population of *Myotis lucifugus* affected in white-nose syndrome. *G3 Genes | Genomes | Genetics*, **10**, 2007-2020. doi:10.1534/g3.119.400966

Liu M, Gao T, Sakurai Y, Jia N, Zhao L, Du X, Jiang Q & Lu Z (2011) Mitochondrial DNA control region diversity and population structure of Pacific herring (*Clupea pallasii*) in the Yellow Sea and the Sea of Japan. *Chinese Journal of Oceanology and Limnology*, **29**, 317-325. doi:10.1007/s00343-011-0008-8

Lonsinger RC, Adams JR & Waits LP (2018) Evaluating effective population size and genetic diversity of a declining kit fox population using contemporary and historical specimens. *Ecology and Evolution*, **8**, 12011-12021. doi:10.1002/ece3.4660

Lounsberry ZT, Almeida JB, Lanctot RB, Liebezeit JR, Sandercock BK, Strum KM, Zack S & Wisely SM (2014) Museum collections reveal that buff-breasted sandpipers (*Calidris subruficollis*) maintained mtDNA variability despite large population declines during the past 135 years. *Conservation Genetics*, **15**, 1197-1208. doi:10.1007/s10592-014-0611-2

Lucentini L, Palomba A, Gigliarelli L, Sgaravizzi G, Lancioni H, Lanfaloni L, Naali M & Panara F (2009) Temporal changes and effective population size of an Italian isolated and supportive-breeding managed northern pike (*Esox Lucius*) population. *Fisheries Research*, **96**, 139-147. doi:10.1016/j.fishres.2008.10.007

Lukoschek V (2018) Population declines, genetic bottlenecks and potential hybridization in sea snakes on Australia's Timor Sea reefs. *Biological Conservation*, **225**, 66-79. doi:10.1016/j.biocon.2018.06.018

Maes GE, Pujolar JM, Hellemans B & Volckaert FAM (2006) Evidence for isolation by time in the European eel (*Anguilla anguilla* L.). *Molecular Ecology*, **15**, 2095-2107. doi:10.1111/j.1365-294X.2006.02925.x

Mamoozadeh NR, Graves JE & McDowell JR (2020) Genome-wide SNPs resolve spatiotemporal patterns of connectivity within striped marlin (*Kajikia audax*), a broadly distributed and highly migratory pelagic species. *Evolutionary Applications*, **13**, 677-698. doi:10.1111/eva.12892

Martin AP (2010) The conservation genetics of Ash Meadows pupfish populations. I. The Warm Springs pupfish *Cyprinodon nevadensis pectoralis*. *Conservation Genetics*, **11**, 1847-1857. doi:10.1007/s10592-010-0077-9

Martínez-Cruz B, Godoy JA & Negro JJ (2007) Population fragmentation leads to spatial and temporal genetic structure in the endangered Spanish imperial eagle. *Molecular Ecology*, **16**, 477-486. doi:10.1111/j.1365-294X.2007.03147.x

Mathieu-Bégné E, Loot G, Chevalier M, Paz-Vinas I & Blanchet S (2019) Demographic and genetic collapses in spatially structured populations: Insights from a long-term survey in wild fish metapopulations. *OIKOS*, **128**, 196-207. doi:10.1111/oik.05511

Matocq MD & Villablanca FX (2001) Low genetic diversity in an endangered species: Recent or historic pattern? *Biological Conservation*, **98**, 61-68. doi:10.1016/S00006-3207(00)00142-7

McBride MC, Hasselman DJ, Willis TV, Palkovacs EP & Bentzen P (2015) Influence of stocking history on the population genetic structure of anadromous alewife (*Alosa pseudoharengus*) in Maine rivers. *Conservation Genetics*, **16**, 1209-1223. doi:10.1007/s10592-015-0733-1

McElroy TC, Kandl KL & Trexler JC (2011) Temporal population genetic structure of eastern mosquitofish in a dynamic aquatic landscape. *Journal of Heredity*, **102**, 678-687. doi:10.1093/jhered/esr088

McKeown NJ, Arkhipkin AI & Shaw PW (2015) Integrating genetic and otolith microchemistry data to understand population structure in the Patagonian hoki (*Macruronus magellanicus*). *Fisheries Research*, **164**, 1-7. doi:10.1016/j.fishres.2014.10.004

Mikle N, Graves TA, Kovach R, Kendall KC & Macleod AC (2016) Demographic mechanisms underpinning genetic assimilation of remnant groups of a large carnivore. *Proceedings of the Royal Society B: Biological Sciences*, **283**, 20161467. doi:10.1098/rspb.2016.1467

Mondol S, Bruford MW & Ramakrishnan U (2013) Demographic loss, genetic structure and the conservation implications for Indian tigers.

Proceedings of the Royal Society B: Biological Sciences, **280**, 1762. doi:10.1098/rspb.2013.0496

Morris K, Austin JJ & Belov K (2013) Low major histocompatibility complex diversity in the Tasmanian devil predates European settlement and may explain susceptibility to disease epidemics. *Biology Letters*, **9**, 20120900. doi:10.1098/rsbl.2012.0900

Morrison CE, Johnson RN, Grueber CE & Hogg CJ (2020) Genetic impacts of conservation management actions in a critically endangered parrot species. *Conservation Genetics*, **21**, 869-877. doi:10.1007/s10592-020-01292-4

Moule H, Chaplin K, Bray RD, Miller KA, Thompson MB & Chapple DG (2015) A matter of time: Temporal variation in the introduction history and population genetic structuring of an invasive lizard. *Current Zoology*, **61**, 456-464. doi:10.1093/czoolo/61.3.456

Moulton LL, Vallender R, Artuso C & Koper N (2017) The final frontier: Early-stage genetic introgression and hybrid habitat use in the northwestern extent of the Golden-winged Warbler breeding range. *Conservation Genetics*, **18**, 1481-1487. doi:10.1007/s10592-017-0989-8

Muñoz-Fuentes V, Green AJ, Negro JJ & Sorenson MD (2005) Population structure and loss of genetic diversity in the endangered white-head duck, *Oxyura leucocephala*. *Conservation Genetics*, **6**, 999-1015. doi:10.1007/s10592-005-9093-6

Nichols HJ, Jordan NR, Jamie GA, Cant MA & Hoffman JI (2012) Fine-scale spatiotemporal patterns of genetic variation reflect budding dispersal coupled with strong natal philopatry in a cooperatively breeding mammal. *Molecular Ecology*, **21**, 5348-5362. doi:10.1111/mec.12015

Nishizawa H, Joseph J & Chong YK (2016) Spatio-temporal patterns of mitochondrial DNA variation in hawksbill turtles (*Eretmochelys imbricata*) in Southeast Asia. *Journal of Experimental Marine Biology and Ecology*, **474**, 164-170. doi:10.1016/j.jembe.2015.10.015

Nunziata SO, Scott DE & Lance SL (2015) Temporal genetic and demographic monitoring of pond-breeding amphibians in three contrasting population systems. *Conservation Genetics*, **16**, 1335-1344. doi:10.1007/s10592-015-0743-z

Osborne MJ, Carson EW & Turner TF (2012) Genetic monitoring and complex population dynamics: Insights from a 12-year study of the Rio Grande silvery minnow. *Evolutionary Applications*, **5**, 553-574. doi:10.1111/j.1752-4571.2011.00235.x

Osborne MJ, Davenport SR, Hoagstrom CW & Turner TF (2010) Genetic effective size, N_e, tracks density in a small freshwater cyprinid, Pecos bluntnose shiner (*Notropis simus pecosensis*). *Molecular Ecology*, **19**, 2832-2844. doi:10.1111/j.1365-294X.2010.04695.x

Osborne MJ, Pilger TJ, Lusk JD & Turner TF (2017) Spatio-temporal variation in parasite communities maintains diversity at the major histocompatibility complex class IIβ in the endangered Rio Grande silvery minnow. *Molecular Ecology*, **26**, 471-489.

doi:10.1111/mec.13936

Ozerov MY, Veselov AE, Lumme J & Primmer CR (2013) Temporal variation of genetic composition in Atlantic salmon populations from the Western White Sea Basin: Influence of anthropogenic factors? *BMC Genetics*, **14**, 88. doi:10.1186/1471-2156-14-88

Padró J, Lambertucci SA, Perrig PL & Pauli JN (2020) Andean and California condors possess dissimilar genetic composition but exhibit similar demographic histories. *Ecology and Evolution*, **10**, 13011-13021. doi:10.1002/ece3.6887

Papetti C, Zane L, Bortolotto E, Bucklin A & Patarnello T (2005) Genetic differentiation and local temporal stability of population structure in the euphausiid *Meganyctiphanes norvegica*. *Marine Ecology Progress Series*, **289**, 225-235. doi:10.3354/meps289225

Pavesi L, De Matthaeis E, Tiedemann R & Ketmaier V (2011) Temporal population genetics and COI Phylogeography of the sandhopper *Macarorchestia remyi* (Amphipoda: Talitridae). *Zoological Studies*, **50**, 220-229.

Perera OP, Fescemyer HW, Fleischer SJ & Abel CA (2020) Temporal variation in genetic composition of migratory *Helicoverpa zea* in peripheral populations. *Insects*, **11**, 463. doi:10.3390/insects11080463

Pérez-Portela R, Turon X & Bishop JDD (2012) Bottlenecks and loss of genetic diversity: Spatio-temporal patterns of genetic structure in an ascidian recently introduced in Europe. *Marine Ecology Progress Series*, **451**, 93-105. doi:10.3354/meps09560

Perrine JD, Pollinger JP, Sacks BN, Barrett RH & Wayne RK (2007) Genetic evidence for the persistence of the critically endangered Sierra Nevada red fox in California. *Conservation Genetics*, **8**, 1083-1095. doi:10.1007/s10592-006-9265-z

Pertoldi C, Barker SF, Madsen AB, Jørgensen H, Randi E, Muñoz J, Baagoe HJ & Loeschcke V (2008a) Spatio-temporal population genetics of the Danish pine marten (*Martes martes*). *Biological Journal of the Linnean Society*, **93**, 457-464. doi:10.1111/j.1095-8312.2007.00892.x

Pertoldi C, Muñoz J, Madsen AB, Barker JSF, Andersen DH, Baagøe HJ, Birch M & Loeschcke V (2008b) Genetic variability in the mitochondrial DNA of the Danish Pine marten. *Journal of Zoology*, **276**, 168-175. doi:10.1111/j.1469-7998.2008.00432.x

Petersen SD, Manseau M & Wilson PJ (2010) Bottlenecks, isolation, and life at the northern range limit: Peary caribou on Ellesmere Island, Canada. *Journal of Mammalogy*, **91**, 698-711. doi:10.1644/09-MAMM-A-231.1

Pfau RS, Goetze JR, Martin RE, Matocha KG & Nelson AD (2019) Spatial and temporal genetic diversity of the Texas kangaroo rat, *Dipodomys elator* (Rodentia: Heteromyidae). *Journal of Mammalogy*, **100**, 1169-1181. doi:10.1093/jmammal/gyz090

Pichler FB & Baker CS (2000) Loss of genetic diversity in the endemic Hector's dolphin due to fisheries-related mortality. *Proceedings of the*

Royal Society B: Biological Sciences, **267**, 97-102. doi:10.1098/rspb.2000.0972

Piggott MP, Banks SC, MacGregor C & Lindenmayer DB (2018) Population genetic patterns in an irruptive species, the long-nosed bandicoot (*Perameles nasuta*). *Conservation Genetics*, **19**, 655-663. doi:10.1007/s10592-017-1044-5

Pilot M, Dabrowski MJ, Jancewicz E, Schtickzelle N & Gliwicz (2010) Temporally stable genetic variability and dynamic kinship structure in a fluctuating population of the root vole *Microtus oeconomus*. *Molecular Ecology*, **19**, 2800-2812. doi:10.1111/j.1365-294X.2010.04692.x

Pita A, Pérez M, Cerviño S & Presa P (2011) What can gene flow and recruitment dynamics tell us about connectivity between European hake stocks in the Eastern North Atlantic? *Continental Shelf Research*, **31**, 376-387. doi:10.1016/j.csr.2010.09.010

Pita A, Pérez M, Velasco F & Presa P (2017) Trends in the genetic effective population size in the Southern stock of the European hake. *Fisheries Research*, **191**, 108-119. doi:10.1016/j.fishres.2017.02.022

Ploshnitsa AI, Goltsman ME, Macdonald DW, Kennedy LJ & Sommer S (2012) Impact of historical founder effects and a recent bottleneck on MHC variability in Commander Arctic foxes (*Vulpes lagopus*). *Ecology and Evolution*, **2**, 165-180. doi:10.1002/ece3.42

Potvin DA, Parris KM, Date KLS, Keely CC, Bray RD, Hale J, Hunjan S, Austin JJ & Melville J (2017) Genetic erosion and escalating extinction risk in frogs with increasing wildfire frequency. *Journal of Applied Ecology*, **54**, 945-954. doi:10.1111/1365-2664.12809

Poulsen NA, Hemmer-Hansen J, Loesckcke V, Carvalho GR & Nielsen EE (2011) Microgeographical population structure and adaptation in Atlantic cod *Gadus morhua*: Spatio-temporal insights from gene-associated DNA markers. *Marine Ecology Progress Series*, **436**, 231-243. doi:10.3354/meps09246

Poulsen NA, Nielsen EE, Schierup MH, Loeschcke V & Grønkjær P (2006) Long-term stability and effective population size in North Sea and Baltic Sea cod (*Gadus morhua*). *Molecular Ecology*, **15**, 321-331. doi:10.1111/j.1365-294X.2005.02777.x

Priest MA, Halford AR & McIlwain JL (2012) Evidence of stable genetic structure across a remote island archipelago through self-recruitment in a widely dispersed coral reef fish. *Ecology and Evolution*, **2**, 3195-3213. doi:10.1002/ece3.260

Pukk L, Kuparinen A, Järv L, Gross R & Vasemägi A (2013) Genetic and life-history changes associated with fisheries-induced population collapse. *Evolutionary Applications*, **6**, 749-760. doi:10.1111/eva.12060

Quinn CB, Alden PB & Sacks BBN (2019) Noninvasive sampling reveals short-term genetic rescue in an insular red fox population. *Journal of Heredity*, **110**, 559-576. doi:10.1093/jhered/esz024

Rangel J, Traver B, Stoner M, Hatter A, Trevelline B, Garza C, Shepherd T, Seeley TD & Wenzel J (2020) Genetic diversity of wild and managed honey bees (*Apis mellifera*) in Southwestern Pennsylvania, and prevalence of the microsporidian gut pathogens *Nosema ceranae* and *N. apis*. *Apidologie*, **51**, 802-814. doi:10.1007/s13592-020-00762-5

Reding DM, Freed LA, Cann RL & Fleischer RC (2010) Spatial and temporal patterns of genetic diversity in an endangered Hawaiian honeycreeper, the Hawaii Akepa (*Loxops coccineus coccineus*). *Conservation Genetics*, **11**, 225-240. doi:10.1007/s10592-009-0025-8

Rhode C, Bester-van der Merwe AE & Roodt-Wilding R (2017) An assessment of spatio-temporal genetic variation in the South African abalone (*Haliotis midae*), using SNPs: Implications for conservation management. *Conservation Genetics*, **18**, 17-31. doi:10.1007/s10592-016-0879-5

Riquet F, Lieutard-Haag C, Serluca G, Woodall L, Claude J, Louisy P & Bierne N (2019) Effective population size and heterozygosity-fitness correlations in a population of the Mediterranean lagoon ecotype of long-snouted seahorse *Hippocampus guttulatus*. *Conservation Genetics*, **20**, 1281-1288. doi:10.1007/s10592-019-01210-3

Roberts DG, Gray CA, West RJ & Ayre DJ (2011) Temporal stability of a hybrid swarm between the migratory marine and estuarine fishes *Acanthopagrus australis* and *A. butcheri*. *Marine Ecology Progress Series*, **421**, 199-204. doi:10.3354/meps08901

Robins JH, Miller SD, Russell JC, Harper GA, Fewster RM (2016) Where did the rats of Big South Cape Island come from? *New Zealand Journal of Ecology*, **40**, 229-234. doi:10.20417/nzjecol.40.26

Rodríguez R, Ramírez O, Valdiosera CE, García N, Alda F, Madurell-Malapeira J, Marmi J, Doadrio I, Willerslev E, Götherström A, Arsuaga JL, Thomas MG, Lalueza-Fox C & Dalén L (2011) 50,000 years of genetic uniformity in the critically endangered Iberian lynx. *Molecular Ecology*, **20**, 3785-3795. doi:10.1111/j.1365-294X.2011.05231.x

Rowe G & Beebee TJC (2004) Reconciling genetic and demographic estimators of effective population size in the Anuran amphibian *Bufo calamita*. *Conservation Genetics*, **5**, 287. doi:10.1023/B:C)GE.0000031145.06421.d3

Rutkowski R, Zawadzka D, Merta D, Stanković A, Jagołkowska P, Suchecka E & Kobielski J (2018) Conservation genetics of the Capercaillie *Tetrao urogallus* in Poland – diversity of mitochondrial DNA in remnant and extinct populations. *Acta Ornithologica*, **52**, 179-196. doi:10.3161/00016454AO2017.52.2.006

Sacks BN, Statham MJ, Perrine JD, Wisely SM & Aubry KB (2010) North American montane red foxes: Expansion, fragmentation, and the origin of the Sacramento Valley red fox. *Conservation Genetics*, **11**, 1523-1539. doi:10.1007/s10592-010-0053-4

Salzburger W, Niederstätter H, Brandstätter A, Berger B, Parson W, Snoeks J & Sturmbauer C (2006) Colour-assortative mating among

populations of *Tropheus moori*, a cichlid fish from Lake Tanganyika, East Africa. *Proceedings of the Royal Society B: Biological Sciences*, **273**, 257-266. doi:10.1098/rspb.2005.3321

Sarno RJ, Jennings DE & Franklin WL (2015) Estimating effective population size of guanacos in Patagonia: An integrative approach for wildlife conservation. *Conservation Genetics*, **16**, 1167-1180. doi:10.1007/s10592-015-0730-4

Schmid S, Neuenschwander S, Pitteloud C, Heckel G, Pajkovic M, Arlettaz R & Alvarez N (2018) Spatial and temporal genetic dynamics of the grasshopper *Oedaleus decorus* revealed by museum genomics. *Ecology and Evolution*, **8**, 1480-1495. doi:10.1002/ece3.3699

Schroeder J, Kentie R, van der Velde M, Hooijmeijer JCEW, Both C, Haddrath O, Baker AJ & Piersma T (2010) Linking intronic polymorphism on the CHD1-Z gene with fitness correlates in black-tailed godwits *Limosa l. limosa*. *International Journal of Avian Science*, **152**, 368-377. doi:10.1111/j.1474-919X.2009.01005.x

Sefc KM, Payne RB & Sorenson MD (2007) Single base errors in PCR products from avian museum specimens and their effect on estimates of historical genetic diversity. *Conservation Genetics*, **8**, 879-884. doi:10.1007/s10592-006-9240-8

Serbezov D, Jorde PE, Bernatchez L, Olsen EM & Vøllestad LA (2012) Life history and demographic determinants of effective/census size ratios as exemplified by brown trout (*Salmo trutta*). *Evolutionary Applications*, **5**, 607-618. doi:10.1111/j.1752-4571.2012.00239.x

Skrbinšek T, Jelenčič M, Waits L, Kos I, Jerina K & Trontelj P (2012) Monitoring the effective population size of a brown bear (*Ursus arctos*) population using new single-sample approaches. *Molecular Ecology*, **21**, 862-875. doi:10.1111/j.1365-294Z.2011.05423.x

Snyder MR & Stepien CA (2017) Genetic patterns across an invasion's history: A test of change versus stasis for the Eurasian round goby in North America. *Molecular Ecology*, **26**, 1075-1090. doi:10.1111/mec.13997

Solorzano CD, Szalanski AL, Owens CB & Stellman CD (2010) Genetic diversity of *Aedes vexans* (Diptera, Culicidae) from New Orleans: Pre- and post-Katrina. *Biochemical Genetics*, **48**, 711-726. doi:10.1007/s10528-010-9354-z

Sonsthagen S, Haughey C, Sexson M, Solovyeva D, Petersen M & Powell A (2020) Temporal variation in genetic structure within the threatened spectacled eider. *Conservation Genetics*, **21**, 175-179. doi:10.1007/s10592-019-01234-9

Sonsthagen SA, Wilson RE &Underwood JG (2017) Genetic implications of bottleneck effects of differing severities on genetic diversity in naturally recovering populations: An example from Hawaiian coot and Hawaiian gallinule. *Ecology and Evolution*, **7**, 9925-9934. doi:10.1002/ece3.3530

Stauffer-Olsen NJ, O'Grady PM & Resh VH (2017) Temporal patterns of genetic diversity in *Baetis tricaudatus* (Ephemeroptera:Baetidae) from

the Russian River, northern California. *Freshwater Science*, **36**, 351-363. doi:10.1086/691973

Stronen AV, Iacolina L, Pertoldi C, Kusza S, Hulva P, Dykyy I, Kojola I & Faurby S (2019) The use of museum skins for genomic analyses of temporal genetic diversity in wild species. *Conservation Genetics Resources*, **11**, 499-503. doi:10.1007/s12686-018-1036-x

Sugimoto T, Aramilev VV, Kerley LL, Nagata J, Miquelle DG & McCullough DR (2014) Noninvasive genetic analyses for estimating population size and genetic diversity of the remaining Far Eastern leopard (*Panthera pardus orientalis*) population. *Conservation Genetics*, **15**, 521-532. doi:10.1007/s10592-013-0558-8

Sun X & Hedgecock D (2017) Temporal genetic change in North American Pacific oyster populations suggests caution in seascape genetics analyses of high gene-flow species. *Marine Ecology Progress Series*, **565**, 79-93. doi:10.33554/meps12009

Suni SS, Bronstein JL & Brosi BJ (2014) Spatio-temporal genetic structure of a tropical bee species suggests high dispersal over a fragmented landscape. *Biotropica*, **46**, 202-209. doi:10.1111/btp.12084

Sutton JT, Robertson BC & Jamieson IG (2015) MHC variation reflects the bottleneck histories of New Zealand passerines. *Molecular Ecology*, **24**, 362-373. doi:10.1111/mec.13039

Taylor SS, Jamieson IG & Wallis GP (2007) Historic and contemporary levels of genetic variation in two New Zealand passerines with different histories of decline. *Journal of Evolutionary Biology*, **20**, 2035-2047. doi:10.1111/j.1420-9101.2007.01362.x

Therkildsen NO, Hemmer-Hansen J, Als TD, Swain DP, Morgan MJ, Trippel EA, Palumbi SR, Meldrup D & Nielsen EE (2013a) Microevolution in time and space: SNP analysis of historical DNA reveals dynamic signatures of selection in Atlantic cod. *Molecular Ecology*, **22**, 2424-2440. doi:10.1111/mec.12260

Therkildsen NO, Hemmer-Hansen J, Hedeholm RB, Wisz MS, Pampoulie C, Meldrup D, Bonanomi S, Retzel A, Olsen SM & Nielsen EE (2013b) Spatiotemporal SNP analysis reveals pronounced biocomplexity at the northern range margin of Atlantic cod *Gadus morhua*. *Evolutionary Applications*, **6**, 690-705. doi:10.1111/eva.12055

Thompson DB, McKelvey K, van Els P, Andrew G, Jacoby-Garrett P, Glenn M, Kallstrom C, Pilgrim KL & Opler PA (2020) Conserve the eco-evolutionary dynamic, not the subspecies: Phenological divergence and gene flow between temporal cohorts of *Euphilotes ancilla* endemic to southern Nevada. *Conservation Genetics*, **21**, 341-357. doi:10.1007/s10592-020-01254-w

Thorburn J, Jones R, Neat F, Pinto C, Bendall V, Hetherington S, Bailey DM, Leslie N & Jones C (2018) Spatial versus temporal structure: Implications of inter-haul variation and relatedness in the North-east Atlantic spurdog *Squalus acanthias*. *Aquatic Conservation – Marine and Freshwater Ecosystems*, **28**, 1167-1180. doi:10.1002/aqc.2922

Tokarska M, Kawałko A, Wójcik JM & Pertoldi C (2009) Genetic variability in the European bison (*Bison bonasus*) population from Białowieża forest over 50 years. *Biological Journal of the Linnean Society*, **97**, 801-809. doi:10.1111/j.1095-8312-2009.01203.x

Tseng M, Kao H, Hung Y & Lee T (2012) A study of genetic variations, population size, and population dynamics of the catadromous Japanese eel *Anguilla japonica* (Pisces) in northern Taiwan. *Hydrobiologia*, **683**, 203-216. doi:10.1007/s10750-011-0958-z

Turner TF, Osborne MJ, McPhee MV & Kruse CG (2015) High and dry: Intermittent watersheds provide a test case for genetic response of desert fishes to climate change. *Conservation Genetics*, **16**, 399-410. doi:10.1007/s10592-014-0666-0

Underwood JN, Richards ZT, Miller KJ, Puotinen ML & Gilmour JP (2018) Genetic signatures through space, time and multiple disturbances in a ubiquitous brooding coral. *Molecular Ecology*, **27**, 1586-1602. doi:10.1111/mec.14559

Vallianatos M, Lougheed SC &Boag PT (2002) Conservation genetics of the loggerhead shrike (*Lanius ludovicianus*) in central and eastern North America. *Conservation Genetics*, **3**, 1-13. doi:10.1023/A:1014232326576

Valtonen M, Palo JU, Ruokonen M, Kunnasranta M & Nyman T (2012) Spatial and temporal variation in genetic diversity of an endangered freshwater seal. *Conservation Genetics*, **13**, 1231-1245. doi:10.1007/s10592-012-0367-5

van Schaik J, Dekeukeleire D & Kerth G (2015) Host and parasite life history interplay to yield divergent population genetic structures in two ectoparasites living on the same bat species. *Molecular Ecology*, **24**, 2324-2335. doi:10.1111/mec.13171

van Schaik J, Kerth G, Bruyndonckx N & Christie P (2014) The effect of host social system on parasite population genetic structure: Comparative population genetics of two ectoparasitic mites and their bat hosts. *BMC Evolutionary Biology*, **14**, 18. doi:10.1186/1471-2148-14-18

Van Wormhoudt A (2015) Seasonal and cyclical changes in genetic composition of the marine intertidal rock pool copepod *Tigriopus brevicornis*. *Biochemical Genetics*, **53** ,79-92. doi:10.1007/s10528-015-9674-0

Vega R, Vázquez-Domínguez E, White TA, Valenzuela-Galván D & Searle JB (2017) Population genomics applications for conservation: The case of the tropical dry forest dweller *Peromyscus melanophrys*. *Conservation Genetics*, **18**, 313-326. doi:10.1007/s10592-016-0907-5

Vilaça ST, Redondo RAF, Lins LV & Santos FR (2012) Remaining genetic diversity in Brazilian Merganser (*Mergus octosetaceus*). *Conservation Genetics*, **13**, 293-298. doi:10.1007/s10592-011-0262-5

Volkmann LA, Statham MJ, Mooers AØ & Sacks BN (2015) Genetic distinctiveness of red foxes in the Intermountain West as revealed through expanded mitochondrial sequencing. *Journal of Mammalogy*, **96**, 297-307. doi:10.1093/jmammal/gyv007

Waits ER, Bagley MJ, Blum MJ, McCormick FH & Lazorchak JM (2008) Source-sink dynamics sustain central stonerollers (*Campostoma anomalum*) in a heavily urbanized catchment. *Freshwater Biology*, **53**, 2061-2075. doi:10.1111/j.1365-2427.2008.02030.x

Waldman J, Atler SE, Peterson D, Maceda L, Roy N & Wirgin I (2019) Contemporary and historical effective population sizes of Atlantic sturgeon *Acipenser oxyrinchus oxyrinchus*. *Conservation Genetics*, **20**, 167-184. doi:10.1007/s10592-018-1121-4

Walsh J, Shriver WG, Correll MD, Olsen BJ, Elphick CS, Hodgman TP, Rowe RJ, O'Brien KM & Kovach AI (2017) Temporal shifts in the saltmarsh – Nelson's sparrow hybrid zone revealed by replicated demographic and genetic surveys. *Conservation Genetics*, **18**, 453-466. doi:10.1007/s10592-016-0920-8

Wells CP, Lavretsky P, Sorenson MD, Peters JL, DaCosta JM, Turnbull S, Uyehara KJ, Malachowski CP, Dugger BD, Eadie JM & Engilis Jr. A (2019) Persistence of an endangered native duck, feral mallards, and multiple hybrid swarms across the Hawaiian Islands. *Molecular Ecology*, **28**, 5203-5216. doi:10.1111/mec.15286

White LC, Moseby KE, Thomson VA, Donnellan SC & Austin JJ (2018) Long-term genetic consequences of mammal reintroductions into an Australian conservation reserve. *Biological Conservation*, **219**, 1-11. doi:10.1016/j.biocon.2017.12.038

Wolf S, Toev T, Moritz RLV & Moritz RFA (2012) Spatial and temporal dynamics of the male effective population size in bumblebees (Hymenoptera: Apidae). *Population Ecology*, **54**, 115-124. doi:10.1007/s10144-011-0285-2

Xie J & Zhang Z (2006) Genetic diversity decreases as population density declines: Implications of temporal variation in mitochondrial haplotype frequencies in a natural population of *Tscherskia triton*. *Integrative Zoology*, **1**, 188-193. doi:10.1111/j.1749-4877.2006.00035.x
