# Supplementary material for: The practice and promise of temporal genomics for measuring evolutionary responses to global change
Source: Mol Ecol Resour. 2023 Apr 2;25(5):e13789. doi: 10.1111/1755-0998.13789 (PMC12142728; doi:10.1111/1755-0998.13789)
Supplement: Supplementary file 3 — Appendix S2 [file MEN-25-e13789-s002.pdf]

The practice and promise of temporal genomics for measuring evolutionary responses to global change

René D. Clark, Katrina A. Catalano, Kyra S. Fitz, Eric Garcia, Kyle E. Jaynes, Brendan N. Reid, Allyson Sawkins, Anthony A. Snead, John C. Whalen, Malin L. Pinsky

SUPPLEMENTAL METHODS

A Web of Science basic search was conducted on February 16th, 2021 individually for 12 keywords (Table 1) in all Web of Science search fields. Web of Science result files are limited to 500 entries; therefore, files were concatenated in R v 4.0.3 (R Core Team, 2019) resulting in 68,222 entries (Table 1). No files were added to the database after the initial search. The database was filtered to remove duplicates, non-journal entries (e.g., conference abstracts, review papers, book chapters), and articles published before the year 2000. Because this systematic literature review is focused on empirical temporal genomic studies in wild animal populations that investigate contemporary changes occurring after the year 1800 CE, the database underwent additional filtering by research area field to remove irrelevant results. The database was filtered to only include pertinent research areas (Table 2); however, an entry may have more than one research area. Therefore, papers were removed if the research area included an irrelevant discipline (Table 2). After filtering by research area, studies focused on ancient populations (before 1800 CE), viruses, and hominins remained. To remove more non-target results, additional automated filtering by the source title, Web of Science categories, author keywords, and article title was completed (Table 2). The final database after filtering in R included 11,187 articles (Table 1). Next, the database of 11,187 articles was further filtered by reading the title and abstract of each article to assess their relevance. This reduced the database down to 611 articles.

Once the title and abstract filtering was complete, each paper's main text and supplementary material were reviewed as well, resulting in 218 papers included in our final database (Table 1). During the final review, each article was classified by subject (adaptation, connectivity, genetic diversity, population size). However, studies often examine more than one topic; therefore, articles were classified by the most prominent subject with additional categories listed in decreasing order of focus. Studies were further categorized by the study organism’s system (terrestrial, freshwater, marine, and other) and its taxonomic level of class. To facilitate a holistic review of the field, data relating to the study design, environmental processes, and molecular protocols were collected for each paper. For study design, this included the country & locations sampled, the years sampled, the number of individuals sampled at each time point, the generation length of the organism studied, and whether the study used museum/archived samples or samples that were entirely collected by the authors themselves. For environmental processes, this included whether the authors were investigating change due to natural or anthropogenic forces, the specific driver of change (invasive species, environmental variation, natural disaster, habitat loss, human exploitation, climate change, disease, competition), and the length of the driver (whether it occurred over an acute or chronic period of time). Finally, for molecular processes, this included the type of genomic marker, the type of tissue DNA was extracted from,

preservation and DNA extraction methods, the type of sequencing platform, and the library preparation methods.

Table 1. The keywords used in the literature search with the number of results returned from Web of Science on February 16th, 2021. Quotation marks indicate that all words in the phrase must appear in the order entered, while phrases not surrounded by quotation marks dictate the words can appear in any order or location. Numbers in columns indicate the number of results associated with each keyword term that remained after the initial search, as well as the 3 subsequent rounds of filtering.

	Number of Results			
**Keyword**	**Initial**	**Post-automatic filter**	**Post-title/abstract filter**	**Post–final filter**
"temporal genomics"	10	0	0	0
temporal genomics	2,282	120	9	6
hdna	109	2	0	0
"historical dna"	110	0	0	0
historical dna	20,812	5,270	190	61
adna	964	52	11	1
"ancient dna"	4,627	0	0	0
ancient dna	11,601	1,531	119	30
"museum dna"	25	0	0	0
museum dna	21,880	3,444	80	22
"temporal genetics"	6	0	0	0
temporal genetics	5,796	768	202	98
**Total**	**68,222**	**11,187**	**611**	**218**

Table 2. The filtering terms used to reduce our database to relevant entries. Filtering terms were extracted directly from the Web of Science results. Terms followed by a dash indicate that the term may not be a word but was used to remove entries that contain any subsequent variation. An asterisk signifies that the term was used with and without capitalization. Only terms found within our database were included; therefore, not all irrelevant terms were used.

Decision	Web of Science Field	Filtering Term			
Include	Research Area	Zoology<br>Ecology<br>Genetic	Fisheries<br>Marine<br>Genomic	Freshwater<br>Evolution<br>Entomology	Biodiversity<br>Oceanography<br>Conservation

Exclude	Research Area	Forestry<br>Energy<br>Cell<br>Anatomy<br>Public<br>Physics<br>Plant<br>Mycology	Agriculture<br>Psychiatry<br>Oncology<br>Medicine<br>Obstetrics<br>Virology<br>Cardiology<br>Toxicology	Veterinary<br>Immunology<br>Sociology<br>Chemistry<br>Microbiology<br>Paleontology<br>Physiology<br>Psychology	Engineering<br>Neurosciences<br>Reproductive<br>Anthropology<br>Archaeology<br>Endocrinology<br>Developmental<br>Ophthalmology
Exclude	Source Title	Human	Medical	Forensic	Clinical
Exclude	Web of Science Category	Clinical	Geosciences		
Exclude	Author Keywords	Paleo-<br>Virus	Bacteria<br>Virology	Medicine<br>Archaeology	Anthropology
Exclude	Article Title	*Domestic<br>archaeolo-	*Homo	*Neanderthal	Archeolo-
